# DESMAN: a new tool for de novo extraction of strains from metagenomes

**DOI:** 10.1186/s13059-017-1309-9

**Published:** 2017-09-21

**Authors:** Christopher Quince, Tom O. Delmont, Sébastien Raguideau, Johannes Alneberg, Aaron E. Darling, Gavin Collins, A. Murat Eren

**Affiliations:** 1 Warwick Medical School, University of Warwick, Gibbet Hill Road, Coventry, CV4 7AL UK; 2 Department of Medicine, University of Chicago, Chicago, USA; 3 KTH Royal Institute of Technology, Science for Life Laboratory, School of Biotechnology, Division of Gene Technology, Stockholm, Sweden; 4 The ithree Institute, University of Technology Sydney, Sydney, Australia; 5 University of Glasgow, Glasgow, UK; 6 Microbial Ecophysiology Laboratory, School of Natural Sciences and Ryan Institute, National University of Ireland, Galway, Ireland; 7 Marine Biological Laboratory, Josephine Bay Paul Center for Comparative Molecular Biology and Evolution, Woods Hole, USA

**Keywords:** Metagenomes, Strain, Niche

## Abstract

We introduce DESMAN for De novo Extraction of Strains from Metagenomes. Large multi-sample metagenomes are being generated but strain variation results in fragmentary co-assemblies. Current algorithms can bin contigs into metagenome-assembled genomes but are unable to resolve strain-level variation. DESMAN identifies variants in core genes and uses co-occurrence across samples to link variants into haplotypes and abundance profiles. These are then searched for against non-core genes to determine the accessory genome of each strain. We validated DESMAN on a complex 50-species 210-genome 96-sample synthetic mock data set and then applied it to the Tara Oceans microbiome.

**Electronic supplementary material:**

The online version of this article (doi:10.1186/s13059-017-1309-9) contains supplementary material, which is available to authorized users.

## Background

Metagenomics, the direct sequencing of DNA extracted from an environment, offers a unique opportunity to study whole microbial communities in situ. The majority of contemporary metagenomics studies use shotgun sequencing, where DNA is fragmented prior to sequencing with short reads, of the order of hundreds of base pairs (bps). To realise the potential of metagenomics fully, methods capable of resolving both the species and the strains present in this data are needed. Reference-based solutions for strain identification have been developed [1, 2] but for the vast majority of microbial species, comprehensive strain-level databases do not exist. This situation is unlikely to change, particularly given the great diversity of microbes that are elusive to standard cultivation techniques [3]. This motivates de novo strategies capable of resolving novel variation at high resolution directly from metagenomic data.

It is not usually possible simply to assemble metagenomic reads into individual genomes that provide strain-level resolution. This is because in the presence of repeats (identical regions that exceed the read length), assemblies become uncertain and fragment into multiple contigs [4]. Metagenomes contain many conserved regions between strains. These act effectively as inter-genome repeats, and hence produce highly fragmented assemblies. This is particularly true when multiple samples are co-assembled together. It is possible to bin these contigs into partitions that derive from the same species using sequence composition [5, 6] and more powerfully, the varying coverage of individual co-assembled contigs over multiple samples [7–10]. However, the resulting genome bins, or metagenome-assembled genomes (MAGs), represent aggregates of multiple similar strains. These strains will vary both in the precise sequence of shared genes, when that variation is below the resolution of the assembler, but also in gene complement, because not all genes and hence, contigs will be present in all strains.

Modified experimental approaches can be used to simplify the challenge of metagenomics assembly by reducing individual sample complexity, for example through enrichment cultures that preferentially grow organisms adapted to particular growth conditions [11] or with potentially less bias by selecting small subsets of cells using flow cytometry and sequencing with low-input DNA techniques [12]. The latter has been coupled with the sequencing of a standard whole-community metagenomics sample in a novel binning pipeline, MetaSort [13], which exploits the assembly graph to map the flow cytometry sample sequences onto those from the community metagenome and to extract genomes. However, for the majority of studies that do not perform enrichment or flow cytometry, improved bioinformatics algorithms will be required to resolve strain variation from metagenome data sets.

A number of methods exist that map reads against reference genes or genomes to resolve strain-level variation de novo [14–16]. The most straightforward approach is to take the consensus single-nucleotide polymorphisms (SNPs) in individual samples to be the haplotypes [16, 17]. This cannot, however, resolve mixtures and will entirely miss strains that are not dominant at least somewhere. These shortcomings can be addressed by using the frequency of the variants across multiple samples to resolve de novo strain-level variation and abundances. This is the approach taken in the Lineage algorithm of O’Brien et al. [14] and ConStrains [15]. However, no method has yet been developed that works from assembled contigs, avoiding the need for any reference genomes, and, hence, is applicable to microbial populations that lack cultured representatives. Here, we show that it is possible to combine this principle with contig-binning algorithms and resolve the strain-level variation in MAGs, both in terms of nucleotide variation on core genes and variation in gene complement.

We denote our strategy DESMAN for De novo Extraction of Strains from Metagenomes. We assume that a co-assembly has been performed and the contigs binned into MAGs. Any binning algorithm could be used for this, but here we applied CONCOCT [9]. We also assume that reads have been mapped back onto these contigs as part of this process. To resolve strain variation within a MAG or group of MAGs deriving from a single species, we first identify core genes that are present in all strains as a single copy. In the absence of any reference genomes, these will simply be those genes known to be core for all bacteria and archaea (single-copy core genes or SCGs), e.g. the 36 clusters of orthologous groups of proteins (COGs) identified in [9]. If reference genomes from the same species or related taxa are available, then these can be used to identify further genes that will satisfy the criteria of being present in all strains in a single copy, in which case we denote these as single-copy core species genes (SCSGs). Using the read mappings, we calculate the base frequencies at each position on the SCSGs or SCGs. Next, we determine variant positions using a likelihood ratio test applied to the frequencies of each base summed across samples. We then use the base frequencies across samples on these variant positions to resolve the number of strains present, their abundance and their unique sequence or haplotype at each variant position for each core gene.

**Fig. 1:**
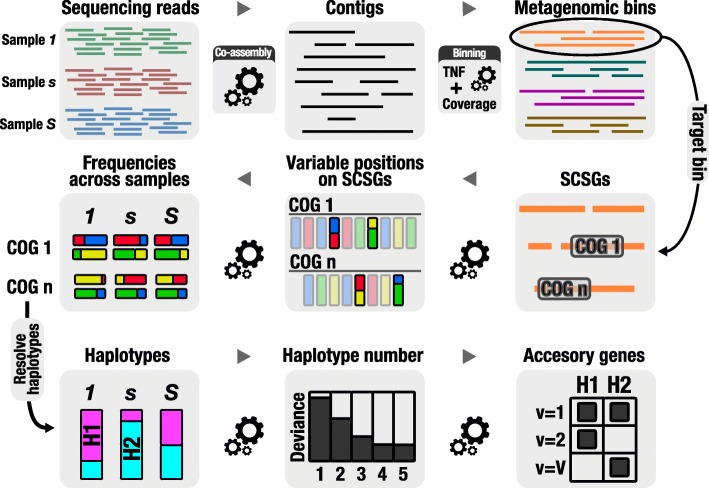
Summary of the DESMAN pipeline. A full description of the statistics and bioinformatics underlying DESMAN is given in ‘Methods’. The software itself is available open source from https://github.com/chrisquince/ DESMAN. COG cluster of orthologous groups of proteins, SCSG single-copy core species gene Tetranucleotide Frequencies (TNF)

The second component of DESMAN is to use this information to determine which accessory genes are present in which strain. From the analysis of core genes, we know how many strains are present and their relative abundances across samples. The signature of relative frequencies across samples associated with each strain will also be observed on the non-core gene variants but, crucially, not all strains will possess these genes and potentially they may be in multiple copies. The relative strain frequencies have to be adjusted, therefore, to reflect these copy numbers. For instance, if a gene is present in just a single copy in one strain, it can have no variants. In addition, the total coverage associated with a gene will also depend on which strains possess that gene being a simple sum of the individual strain coverages. Here, we do not address the multi-copy problem, just gene presence or absence in a strain. We infer these given the observed variant base frequencies and gene coverages across samples whilst keeping the strain signatures fixed at those computed from the SCSGs and SCGs. This also provides a strategy for inferring non-core gene haplotypes on strains. Taken together, these two steps provide a procedure for resolving both strain haplotypes on the core genome and their gene complements entirely de novo from short-read metagenome data. We recommend applying this strategy to genes, but crucially genes called on the assembled contigs. If contig assignments are preferred, the same methodology could be applied directly to the contigs themselves, or a consensus assignment of genes on a contig used to determine its presence or absence in a given strain. The DESMAN pipeline is summarised in Fig. 1.

The advantage of using base frequencies across samples to resolve strains, rather than existing haplotype resolution algorithms that link variants using reads [18], is that it enables us to resolve variation that is less divergent than the reciprocal of the read length and to link strains across contigs. The intuition behind frequency-based strain inference is similar to that of contig binning. The frequencies of variants associated with a strain fluctuate across samples with the abundance of that strain. However, in this case it is necessary to consider that multiple strains may share the same nucleotide at a given variant position. To solve this problem, we develop a full Bayesian model, fitted by a Markov chain Monte Carlo (MCMC) Gibbs sampler, to learn the strain frequencies, their haplotypes and also sequencing error rates. To improve convergence, we initialise the Gibbs sampler using non-negative matrix factorisation (NMF), or more properly non-negative tensor factorisation (NTF), a method from machine learning that is equivalent to the maximum likelihood solution [19]. Our approach is like the Lineage algorithm developed by O’Brien et al. [14], except that they have a simpler noise model but a more complex prior for the strain haplotypes derived from an underlying phylogenetic tree. Both approaches differ from the heuristic strategy for strain inference used in ConStrains [15]. The full Bayesian approach allows not just a single estimate of the strain haplotypes, but also an estimate of the uncertainty in the predictions through comparison of replicate MCMC runs.

To illustrate the efficacy of the DESMAN pipeline, we first apply it to the problem of resolving *Escherichia coli* strains in metagenomic data sets. *E. coli* has a highly variable genome [20], and while some strains of *E. coli* occur as harmless commensals in the human gut, others can be harmful pathogens. We used a synthetic data set of 64 samples generated from an *in silico* community comprising five *E. coli* strains and 15 other strains commonly found in human gut samples (see Additional file 1: Table S1). Strains in this data set were present in each sample with varying abundances determined by 16S rRNA community profiles obtained from the Human Microbiome Project (HMP) [21]. The reads themselves simulated a typical HiSeq 2500 run. We then applied DESMAN to 53 real faecal metagenome samples from the 2011 Shiga-toxin-producing *E. coli* (STEC) O104:H4 outbreak [22] and validated our ability to resolve the outbreak strain correctly. The results from these analyses were encouraging but the real potential of DESMAN is to resolve strains for environmental populations without any cultured representatives. To validate the effectiveness of DESMAN on more complex communities when only the 36 SCGs are used for haplotype inference, we applied it to an *in silico* synthetic community of 100 species and 210 strains with 96 samples. Having demonstrated that the results are reliable even in this case, we ran DESMAN on the 32 most abundant MAGs from a collection of 957 non-redundant MAGs reconstructed by Delmont et al. from the Tara Oceans project metagenomes [23].

## Results

### Synthetic strain mock

#### Contig binning with CONCOCT

The assembly statistics for this synthetic strain mock are given in Additional file 1: Table S2. CONCOCT clustered the resulting 7,545 contig fragments from these 20 genomes into 19 bins. Additional file 1: Figure S1 compares CONCOCT bins for each contig with the genome from which they originated. This clustering combined shared contigs across *E. coli* strains into bin 6, and the remaining strain-specific contigs were contained in bin 16 (Additional file 1: Figure S1). To extract strains with DESMAN, we first combined bins 6 and 16 to recover the *E. coli* pangenome, which contained 2,028 contigs with a total length of 5,389,019 bp. We then identified coding domains in this contig collection and assigned them to 2,854 COGs, 372 of which matched our 982 SCSGs for *E. coli* (see ‘Identifying core genes in target species’). These 372 SCSGs had a total length of 255,753 bp, and we confirmed that each of them occurred as a single copy in our contig collection.

#### Variant detection

We mapped reads from each sample onto the contig sequences associated with the 372 SCSGs to obtain sample-specific base frequencies at each position. We identified variant positions using the likelihood ratio test defined below (Eq. 2), classifying positions as variants if they had a false discovery rate (FDR) of less than 10^−3^. As an example, Additional file 1: Figure S2 displays the likelihood ratio test values for a single COG (COG0015 or adenylosuccinate lyase) across nucleotide positions, along with true variants as determined from the known genome sequences. Additional file 1: Table S3 reports the confusion matrix comparing the 6,044 predicted variant positions across all 372 SCSGs with the known variants. Our test correctly recalled 97.9% of the true variant positions with a precision of 99.9% (Additional file 1: Table S3). Our analysis missed 125 variant positions, but manual inspection revealed that this is almost entirely due to incorrect mapping rather than the variant discovery algorithm per se.

#### Strain deconvolution

Having identified 6,044 potential variant positions on the 372 SCSGs, we then ran the haplotype deconvolution algorithm with increasing number of strains *G* from three to eight. We ran the Gibbs sampler on 1,000 positions chosen at random with five replicate runs for each *G*. Each run comprised 100 iterations of burn-in followed by 100 samples as discussed below. The runs were initialised using the NTF algorithm with different random initialisations. We generated posterior samples for the strain frequencies and error rates using the 1,000 randomly selected positions. These parameters will apply for all variants; hence, we could then use these samples to assign bases at all positions for the haplotypes. This was done by generating 100 samples following 100 samples of burn-in for these base assignments.

**Fig. 2:**
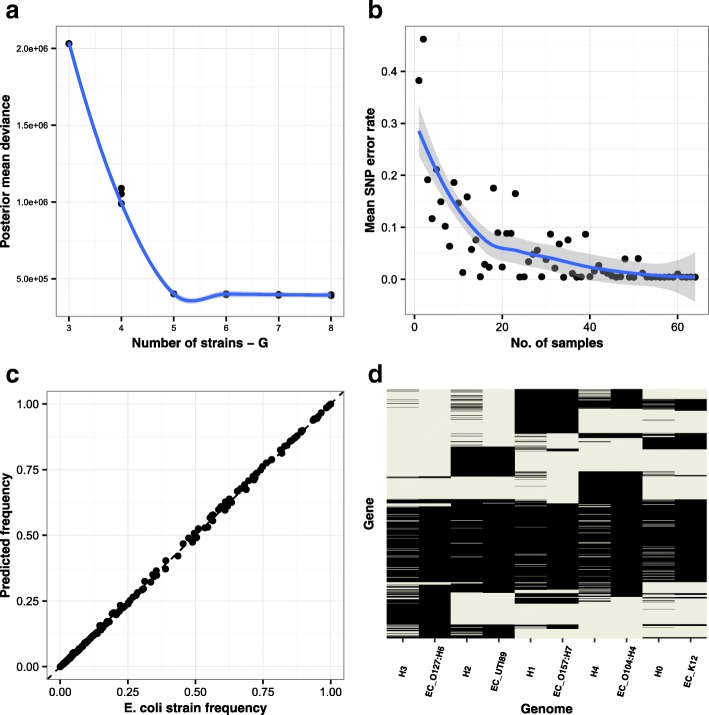
**a** Posterior mean deviance for different strain numbers, *G*, for the synthetic strain mock *Escherichia coli* SCSG positions. We ran five replicates of the Gibbs sampler at each value of *G* on 1,000 random positions from the 6,044 variants identified. **b** SNV accuracy as a function of sample number. The number of incorrectly inferred SNVs averaged across all five strains and 20 replicates of a random subset of the 64 samples. **c** Comparison of true *E. coli* strain frequency vs. DESMAN predictions. We compare the known *E. coli* strain frequencies as relative coverage against the frequencies in each sample of the DESMAN-predicted haplotype it mapped onto (*R* ^2^=0.9998, *p*-value <2.2×10^−16^). **d** Comparison of gene presence inferred for the haplotypes and the known assignment of genes to strain genomes. Gene presence/absence was inferred for the haplotypes using Eq. 8 and compared to known references. Overall accuracy was 95.7%. These results were for the run with *G*=5, which had the lowest posterior mean deviance. *E. coli* *Escherichia coli*, SNP single-nucleotide polymorphism, SNV single-nucleotide variant

Figure 2 a gives the posterior mean deviance, a proxy for model fit, as a function of *G*. We can see from this that the deviance decreases rapidly until *G*=5, after which the curve flattens. In this case, we can easily identify that the number of strains is indeed the five *E. coli* strains present in our mock community. We can now assess how well we can reconstruct the known sequences for *G*=5. Additional file 1: Table S4 compares the posterior mean strain predictions for the run with *G*=5 and lowest posterior mean deviance with the known reference genomes. Each haplotype maps onto a distinct genome with error frequencies varying from 10 to 39 positions out of 6,044, representing error rates from 0.17 to 0.64% of single-nucleotide variant (SNV) positions. The percentage of correctly predicted variable positions averaged over haplotypes was 99.58%.

This level of accuracy is sufficient to broadly resolve strain-level phylogenetic relationships. In Additional file 1: Figure S3, we display the phylogenetic analysis of 62 reference *E. coli* genomes together with the inferred strain sequences constructed using the 372 SCSGs. In four out of five cases, the closest relative to each strain on the tree was the genome actually used to construct the synthetic strain mock. In the one case where it was not, *E. coli* K12, the strain was most closely related to three highly similar K12 strains, including that used in the synthetic community. Fine-scale strain variation smaller than the SNV error rates would not be correctly resolved on this tree but the accuracy is sufficient to place the inferred haplotypes within the major *E. coli* lineages.

#### Comparison to existing algorithms

We also ran the Lineage algorithm from O’Brien et al. [14] on the same mock data. The model was run on the same 1,000 variants selected at random from the 6,044 variant positions we identified. We could not run the full 6,044 variant positions because of run time limitations. Their model also correctly predicted five haplotypes; however, two of these were identical, and matched exactly to the EC_K12 strain. Of the other three predictions, one was only seven SNVs different from EC_O104, yet the other two did not correspond to any of the true genomes. The average accuracy of prediction (the percentage of correctly predicted variable positions mapping each predicted haplotype onto the closest unique reference) was 76.32%. Additional file 1: Table S5 compares the Lineage predictions to the known strains. To provide a completely transparent comparison with DESMAN, we also compare the DESMAN predictions to the known strains on just these 1,000 variant positions in Additional file 1: Table S6. That gave an average accuracy of 99.6%. We were unable to run ConStrains [15] on the same data set, as the program complained that *insufficient coverage* of *E. coli* specific genes was obtained from the MetaPhlAn mapping. This is despite the fact that the *E. coli* coverage across our samples ranged between 37.88 and 432.00, with a median coverage of 244.00, well above the minimum of 10.0 stated to be necessary to run the ConStrains algorithm [15].

#### Effect of sample number on strain inference

To quantify the number of samples necessary for accurate strain inference, for each sample number between 1 and 64 we chose a random subset of samples that had mean strain relative abundances as similar as possible to those in the complete 64. We then ran DESMAN as above but using only these samples. This was done after the variant detection so all positions identified as variants were potentially included in the subsets. We ran 20 replicates of the Gibbs sampler at each sample number and then calculated SNV error rates for these runs, i.e. the fraction of positions at which the inferred SNV differed from the true SNP in the closest matching reference. This was averaged over all five strains and 20 replicates. The results are shown together with the original 64 samples in Fig. 2 b. The SNV error rate starts to increase when the sample number is below about 30; however, the average error is still around 15%, even with just ten samples. In addition, at low sample number, the accuracy is very variable across strains, and typically some of the strains are resolved accurately and others are missed completely.

#### Inference of strain abundances

DESMAN also predicts the frequencies of each strain in each sample. We validated these predictions by comparing with the known frequencies of the *E. coli* genome each inferred strain mapped onto (Additional file 1: Table S4). The relative frequencies predicted by DESMAN are the proportion of coverage deriving from each strain. For the synthetic mock, we specified the relative genome frequency of each strain in each sample; therefore, we had to normalise these by the inverse of the strain genome lengths and renormalise. Thus, the relative strain coverage is

$$\pi_{g,s} = \frac{\pi^{\prime}_{g,s}/L_{g}}{\sum_{h} \pi^{\prime}_{h,s}/L_{h}}, $$

where *L*_*g*_ is the length of genome *g* and $\pi ^{\prime }_{g,s}$ the relative genome frequency. Through this analysis, we obtained an almost exact correspondence between the relative frequencies for all five strains in all 64 samples (see Fig. 2 c). A linear regression of actual values against predictions forced through the origin gave a coefficient of 0.996, an adjusted *R* ^2^=0.9998 and *p*-value <2.2×10^−16^.

#### Run times

Running DESMAN for one choice of strain number, *G*=5, took on average 116.86 min for the synthetic strain mock. This was using one core on an Intel(R) Xeon(R) CPU E7-8850 v2 at 2.30 GHz. There is no parallelisation of the Gibbs sampler at the heart of DESMAN but since replicate MCMC runs and different strain numbers do not communicate, then this is an example of an embarrassingly parallel problem where each run can be performed simultaneously. The run time scales approximately linearly with sample number (see Additional file 1: Figure S4).

#### Gene assignment

To validate the method for non-core gene assignment to strains in DESMAN, we took the posterior mean strain frequencies across samples and the error matrix from the run with *G*=5 that had the lowest posterior mean deviance. These were then used as parameters to infer the presence or absence of each gene in each strain, given their mean gene coverages and the frequencies of variant positions across samples (Eq. 9). Figure 2 d compares these inferences with the known values for each reference genome. We can determine whether a gene is present in a strain genome with an overall accuracy of 94.9%.

### *E. coli* O104:H4 outbreak

#### Assembly, contig binning, core gene identification and variation detection

The results for the synthetic mock community are encouraging, and they demonstrate that in principle DESMAN should be able to resolve strains accurately from mixed populations de novo. However, it can never be guaranteed that performance on synthetic data will be reproduced in the real world. There are always additional sources of noise that cannot be accounted for in simulations. Therefore, for a further test of the algorithm, we applied it to 53 human faecal samples from the 2011 STEC O104:H4 outbreak. Here, we do not know the exact strains present and their proportions but we do know one of the strains, the outbreak strain itself from independent genome sequencing of cultured isolates [24]. Hence, we can test our ability to resolve this particular strain.

In Additional file 1: Table S2, we give the assembly statistics for the *E. coli* O104:H4 outbreak data. We used the CONCOCT clustering results from the original analysis in Alneberg et al. (2014) as our starting point for the strain deconvolution. From the total of 297 CONCOCT bins, we focused on just three, 95% of the contigs in which could be taxonomically assigned to *E. coli*. These bins were denoted as 83,122 and 216 in the original nomenclature, and together they contained 2,574 contigs with a total length of 7,239 kbp. We identified 4,651 COGs in this contig collection, 673 of which matched with the 982 SCSGs that we identified above for *E. coli*. We expect that all core genes should have the same coverage profiles across samples. We can, therefore, compare the coverage of each putative SCSG against the median in that sample. On this basis, we filtered a further 233 of these SCSGs, leaving 440 for the downstream analysis with a total length of 420,220 bp. This is an example of the extra noise arising in real samples. For the synthetic community, this filtering strategy would remove no SCSGs (hence, this is why it was not applied above).

We obtained sample-specific base frequencies at each position by mapping reads from each of the 53 STEC samples onto the contig sequences associated with the 440 SCSGs. In the following analysis, we used only the 20 samples, in which the mean coverage of SCSGs was greater than five. It is challenging to identify variants confidently in samples with less coverage. Aggregating frequencies across samples, we detected 28,435 potential variants (FDR <1.0×10^−3^) on these SCSGs, which were then used in the strain inference algorithm.

#### Strain deconvolution

Using these 20 samples, we ran the strain deconvolution algorithm with increasing numbers of strains *G* from two to ten, like the analysis above, except that for these more complex samples, we used 500 iterations rather than 100 for both the burn-in and sampling phase. Additional file 1: Figure S5 displays the posterior mean deviance as a function of strain number, *G*. From this, we deduce that eight strains are sufficient to explain the data.

#### Strain sequence validation

**Fig. 3:**
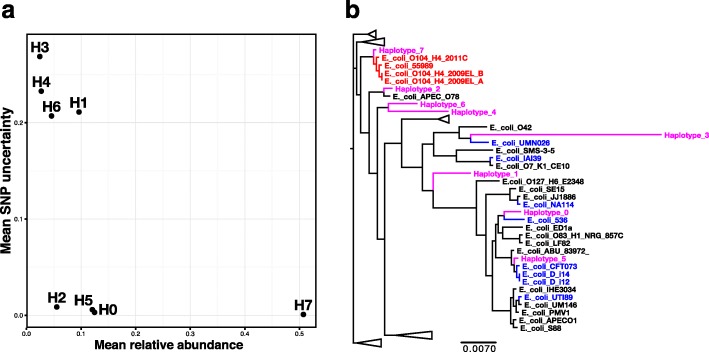
Validation of reconstructed strains for the *Escherichia coli* O104:H4 outbreak. **a** The mean SNV uncertainty, i.e. the proportion of SNVs that a strain differs from its closest match in a replicate run, averaged over all the other replicates. This is shown on the *y*-axis against mean relative abundance across samples on the *x*-axis. **b** Phylogenetic tree constructed for the eight inferred strains found for the *E. coli* O104:H4 outbreak. The SCSGs for the strains and reference genomes were aligned separately using mafft [50], trimmed and then concatenated together. The tree was constructed using FastTree [51]. Inferred strains are shown as magenta, O104:H4 strains in red and uropathogenic *E. coli* in blue. Both results were for the run with *G*=8 that had the lowest posterior mean deviance. SNP single-nucleotide polymorphism, SNV single-nucleotide variant

We selected the replicate run with eight strains that had the lowest posterior mean deviance, i.e. the best overall fit. To determine the reliability of these strain predictions, we compared them with their closest match in the replicate runs. Due to both the random initialisation of the NTF and the stochastic nature of MCMC sampling, strains in replicates are not expected to be identical. However, the consistent emergence of similar strains across replicates increases our confidence in their prediction. Figure 3 a displays the comparison of each strain in the selected run to its closest match in the alternate runs, as the proportion of all SNVs that are identical averaged over positions and all four alternate replicates. This is given on the *y*-axis against mean relative abundance across all samples on the *x*-axis. From this we see that the strains fall into two groups, four relatively low abundance strains with high SNV uncertainties >20% (H1, H3, H4 and H6) and four of varying abundance that we are very confident in, each with uncertainties <1% (H0, H2, H5 and H7). These results are confirmed by Fig. 3 b, where we present a phylogenetic tree constructed from these SCSGs for the eight inferred strains and 62 reference *E. coli* genomes. For example, strain H3 forms a long terminal branch, suggesting that it does not represent a real *E. coli* strain. Similarly, H1, H4 and H6 are not nested within reference strains, whereas, in contrast, the four strains with low SNV uncertainties are placed adjacent to known *E. coli* genomes. In Additional file 1: Table S7, we give the closest matching reference sequence for each strain together with nucleotide substitution rates calculated from this tree. Strain H7 is 99.8% identical to an O104:H4 outbreak strain sequenced in 2011 and H5 is closely related (99.8%) to a clade mostly composed of uropathogenic *E. coli*. In fact, all four strains that we are confident in are within 1% of a reference, whereas none of the other four are.

We then inferred the presence or absence of all 8,566 genes in the three *E. coli* bins for the eight strains using Eq. 9. Strain H7, which matches the outbreak strain on core gene identity, was also closest in terms of accessory gene complement, with 91.8% of the inferred gene predictions identical to the result of mapping genes onto the O104:H4 outbreak strain (Additional file 1: Figure S6). In Additional file 1: Figure S7, we give the relative frequencies for each of the eight inferred strains across the 20 samples with sufficient *E. coli* core genome coverage (>5.0) for strain inference. Here, we have ordered samples associated with STEC by the number of days since the diarrhoeal symptoms first appeared. This variable is marginally negatively associated with the abundance of strain H7, which fits with our identification that it is the 2011 O104:H4 outbreak strain.

### Complex strain mock

#### Contig binning with CONCOCT

The complex strain mock consisted of 210 genomes from 100 species distributed across 96 samples. Half of the species had no strain variation, 20 had two strains, 10 three strains, 10 four strains and 10 five strains (see ‘Methods’). The reads from this mock assembled into 74,580 contig fragments with a total length of 409 Mbp compared to 687 Mbp for all 210 genomes. CONCOCT generated 137 clusters, suggesting some clusters will be aggregates of strains from the same species whereas other species are split across clusters. This was confirmed by comparing the cluster assignments to the known contig species assignments, giving a recall of 86.1% and a precision of 98.2%. This indicates that most clusters contain only one species but some species are fragmented (Additional file 1: Figure S8).

For the complex mock, we decided to model a situation corresponding to studying a novel environment where accurate taxonomic classifications may be impossible and species-specific core gene collections unavailable. We, therefore, applied DESMAN without aggregating clusters and using only the 36 single-copy genes that are core to all prokaryotes (SCGs) for the variant analysis. There were 75 clusters that had at least 75% of these genes in a single copy. These were considered sufficiently high-quality bins for subsequent DESMAN analysis (Additional file 1: Figure S9).

#### Variant detection

**Fig. 4:**
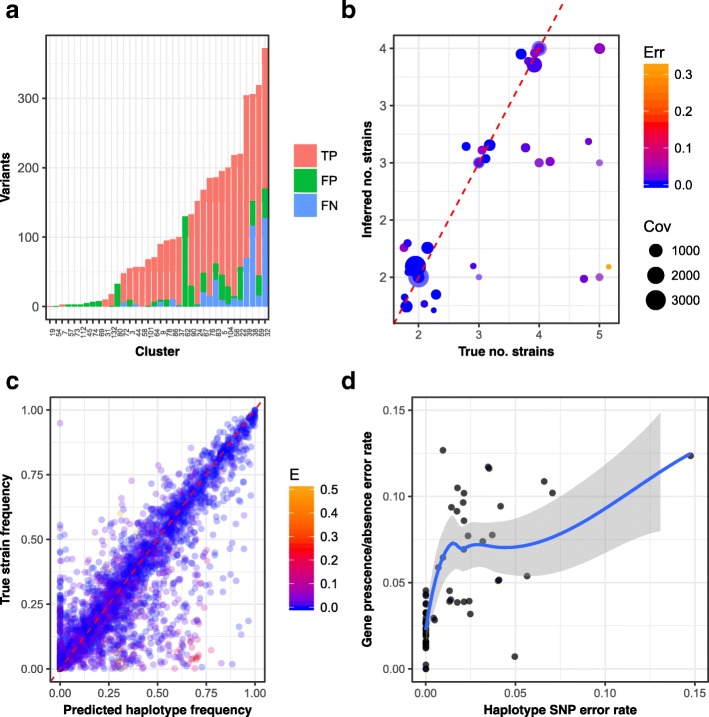
**a** Variant detection for the 75 CONCOCT clusters of complex strain mock that were 75% pure and complete. Here, 36 clusters (shown) had variants, and 27 of these mapped onto multi-strain species enabling us to calculate variants that were present in the species (true positives or TPs), the number detected not in the species (false positives or FPs) and the number we failed to detect (false negatives or FNs). **b** Haplotype inference accuracy. For the 25 75% complete CONCOCT clusters that possessed variants and mapped onto species with strain variation, we plot the true number of strains (*x*-axis) against the inferred number (*y*-axis), with random jitter to distinguish data points. The colour reflects the mean error rate in SNV predictions on single-copy core genes (Err) and the size the total coverage of the cluster (see Additional file 1: Table S8 for actual values). **c** Comparison of the true relative strain frequency and inferred haplotype frequency across the 96 samples for the complex strain mock. The data points are coloured by the SNV error rate (*E*) in the haplotype prediction. (Linear regression of true vs. predicted frequency all: slope = 0.820, adjusted R-squared = 0.741, *p*-value = <2.2×10^−16^; haplotypes with *E*<0.01: slope = 0.853, adjusted R-squared = 0.810, *p*-value <2.2×10^−16^.) **d** Haplotype SNV error vs. gene presence/absence inference error rate. For each of the 67 inferred haplotypes, we give the SNV error rate on single-copy core genes to the closest reference strain against the error rate in the prediction of gene presence/absence in that strain. Cov coverage, Err error, FN false negative, FP false positive, SNP single-nucleotide polymorphism, SNV single-nucleotide variant, TP true positive

We began by filtering the SCGs in each cluster for outliers based on median coverage and then applied variant detection at each position as described below (see ‘Methods’). Following filtering, the median number of SCGs across clusters was reduced from 35 to 30, with a minimum of 19. To determine the true variants for validation, we mapped each cluster to the species that the majority of its contigs derived from and determined exactly which variants were present on the SCGs for those species that had multiple strains (see ‘Methods’). Of the 75 clusters, we predicted variants in 36, including 27 of the 29 that should have exhibited SNVs on the SCGs (see Fig. 4 a). Over those 27 clusters, we predicted a median of 99 variants per cluster, with a minimum of 1 and a maximum of 303. Comparing to the true variant positions, we obtained a mean precision of 92.32% and a mean recall of 91.85%. Here, 25 of the 27 clusters had at least five variants and this subset was used below for haplotype deconvolution. Attempting to deconvolve haplotypes with fewer potential variants than this would be very difficult.

In the two clusters that should have had variants for which none were observed, we missed 4 and 265 real variants. Manual investigation revealed that false negatives in variant detection were often caused by strain variation exceeding the maximum number of differences allowed in a read during mapping or because that SCG had been assembled into multiple contigs. There were nine clusters that should not have had strain variation for which variants were detected. Over these clusters, we predicted a median of nine variants, with five clusters having at least five variants including one cluster with 130 variants. This cluster must have recruited reads from a closely related species or was a contaminated bin to begin with. The results of the SCG filtering and variant detection for each cluster are given in Additional file 2.

#### Haplotype deconvolution

For each of the 25 clusters for which we predicted five or more SNVs and for which multiple strains were present in the assembly, we ran the haplotype deconvolution algorithm with increasing numbers of haplotypes *G* from 1 to 7. The highest variant number was just 303, enabling all variant positions to be used for inference by the Gibbs sampler. We performed ten replicates of each run with 250 iterations of burn-in followed by 250 samples (see ‘Methods’). For each cluster, we then used a combination of the posterior mean deviance and the mean SNV uncertainty to determine the optimal number of haplotypes present using an automated heuristic algorithm (see ‘Methods’). This strategy predicted the correct haplotype number for 18/25 (72%) of the clusters. For 22/25 (88%) of the clusters, the predicted haplotype number was within 1 of the true value (see Additional file 1: Table S8 and Fig. 4 b). The largest number of haplotypes correctly inferred was four. For the nine clusters from single-strain species in which variants were observed incorrectly, we applied haplotype deconvolution to the five clusters with at least five variants. We correctly predicted that a single haplotype was present for three of these clusters, but we inferred two haplotypes in one and three in the final cluster, i.e. there were three false positive haplotype predictions.

Mapping each inferred haplotype onto the closest matching reference, we calculated the fraction of variants incorrectly inferred averaged over all haplotypes in the cluster to obtain a mean SNV error rate. For 15/25 (60%) of the clusters, this was below 1% with a median of 0.25% and a mean of 2.38%, being driven by some highly erroneous inferences. There was no correlation between the error rate and either the number of variants in the cluster or the coverage. However, when we consider each individual strain in all 25 species (79 in total) of which 67 strains (or 84.8%) were detected, we do find a positive relationship between detection and individual strain coverage (Additional file 1: Figure S10, logistic regression *p*-value = 0.0035). We detected every strain that was more than 100 SNPs divergent from its closest relative, which translates into a nucleotide divergence of approximately 0.38% given a mean length for the 36 SCGs of 26.4 Mbp. We were able to detect strains successfully in some clusters using as few as ten SNVs (e.g. Cluster31; see Additional file 1: Table S8).

In summary, across the 75 clusters, which we know should have comprised 133 strains, we inferred five or more SNVs in 30. Applying DESMAN to these, we predicted a total of 75 haplotypes. So our 75 consensus sequences are transformed by DESMAN into 75 haplotypes and 45 consensus sequences for a total of 120 sequences. Of these 75 haplotypes, three were false positives, and of the 67 from true multi-strain clusters, 34 (50.7%) were obtained exactly and 53 (79.1%) were within five SNVs of their closest matching reference.

#### Inference of strain abundances and gene assignments

We compared the inferred relative frequency of each haplotype with the frequency of the closest matching strain (insisting on a one-to-one mapping) across the 96 samples. For the accurately resolved haplotypes, there was a close match (see Fig. 4 c). A linear regression across all strains of true frequency as a function of predicted gave a slope of 0.820 (adjusted R-squared = 0.741, *p*-value <2.2×10^−16^). This suggests a bias towards underestimating the true frequency, which was reduced when only accurately resolved strains (SNV error rate < 1%) were considered (slope = 0.853, adjusted R-squared = 0.810, *p*-value <2.2×10^−16^). Finally, for each haplotype, we inferred the presence or absence of each gene in the cluster, given their mean gene coverages and frequencies of variant positions across samples (Eq. 9). We then compared these predictions to the known assignments of genes (see ‘Methods’) of the strain that the haplotype mapped to. Averaged over all 67 detected haplotypes, the resulting gene prediction accuracy was 94.9% (median 96.26%) and this increased to 97.39% for the 39 haplotypes that we predicted with an SNV error rate less than 1%. There was a strong positive relationship between how accurately the haplotype was resolved as measured by SNV error rate on the SCGs and the error rate in the gene predictions (adjusted R-squared = 0.697, *p*-value <2.2×10^−16^; see Fig. 4 d).

#### Comparison to Lineage algorithm

To provide a comparison to the DESMAN haplotype inference, we ran the Lineage algorithm on the 25 clusters for which five or more variants were present and which mapped onto species with strain variation. For each cluster, we ran 4,000 MCMC iterations of their sampler. The results are given in Additional file 1: Table S8. Overall the results were comparable to DESMAN, but Lineage correctly inferred the correct strain number for only 15 (60%) of the clusters rather than the 18 obtained by DESMAN. The median and mean SCG SNV error rates for the inferred haplotypes in a cluster were also higher at 0.641% and 3.583%, respectively, compared to 0.25% and 2.38% for DESMAN, an increase that was almost significant, when we compared the Lineage and DESMAN error rates across clusters (Kruskal–Wallis paired ANOVA, *p*-value = 0.06). We also compared the Lineage predicted haplotype frequencies with the true strain frequencies as we did above for DESMAN and we obtained a worse correlation between the two (slope = 0.804, adjusted R-squared = 0.6665, *p*-value <2.2×10^−16^), although again the results improved when restricted to haplotypes with SNV error rates <1% (slope = 0.839, adjusted R-squared = 0.7088, *p*-value <2.2×10^−16^).

### Tara Oceans plankton microbiome survey

The Tara Oceans microbiome survey generated 7.2 terabases of metagenomic data from 243 samples across 68 locations from epipelagic and mesopelagic waters around the globe [23]. In the original study, no attempt was made to extract genomes from these sequences and no strain resolution was performed. Recently, Delmont et al. extracted 957 non-redundant MAGs from a subset of 93 of these samples, comprising 61 surface samples and 32 from the deep chlorophyll maximum layer [25]. The MAGs were generated by performing 12 geographically bounded co-assemblies (see Additional file 1: Figure S11), then initial binning of contigs by composition and coverage using CONCOCT, followed by refinement with the Anvi’o interactive interface [10].

We took the 32 most abundant MAGs (total coverage > 100.0) with at least 75% of SCGs as single copy and applied the DESMAN pipeline to resolve their strain diversity. These 32 MAGs derived from six different phyla (four Actinobacteria, six Bacteroidetes, one Candidatus Marinimicrobia, one Chloroflexi, three Euryarchaeota and 17 Proteobacteria).

#### Variant detection

We mapped the reads from the 93 individual samples onto our entire MAG contig collection and then separated out the mappings onto the SCGs for our 32 focal MAGs. We filtered the SCGs for those with outlying coverages (see ‘Methods’). The numbers of SCGs before and after filtering and their total sequence length are given in Additional file 1: Table S10. The median number of SCGs was reduced from 32.5 to 23.5 after filtering. We then ran variant detection on these filtered SCGs. The total number of SNVs detected in each MAG varied from 1 to 2,602 with a median of 359. The observed percentage frequency of SNVs, normalised by the total number of base pairs of the sequence tested, given our minimum detection cut-off of 1%, varied considerably between MAGs, ranging from 0.07 to 12.57% with a median of 2.86%.

The SNV frequency was independent of MAG coverage (Spearman’s *p*-value = 0.84) and the number of samples that the MAG was found in (Spearman’s *p*-value = 0.22). This confirms that we had sufficient coverage to detect all SNVs above the 1% threshold. We observed a negative correlation with genome length (Spearman’s *p*-value = 0.025) and a stronger negative relationship with number of KEGG pathway modules encoded in the MAG (Spearman’s *p*-value = 0.0049; see Additional file 1: Figure S12). This correlation was independent of MAG taxonomic assignment (Kruskal–Wallis ANOVA against phyla *p*-value = 0.1672). We also compared for each MAG the fraction of AT vs. GC base positions for those bases that were not flagged as variants and those that were. There was a significant bias observed for AT positions in non-variant bases (*t* = 2.7616, *p*-value = 0.00958, mean difference = 0.06).

#### Haplotype deconvolution

**Fig. 5:**
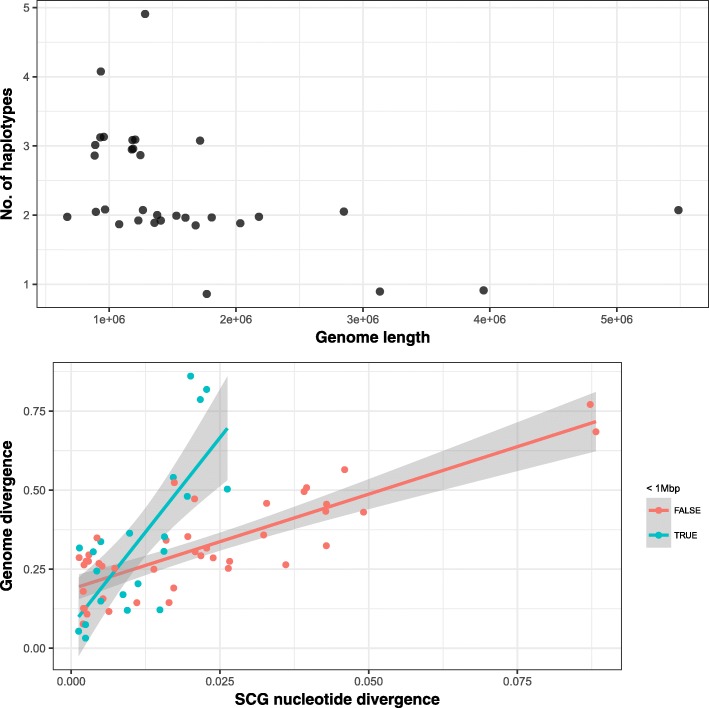
Top panel: Number of haplotypes inferred by DESMAN as a function of MAG genome length. A significant negative correlation was observed (Spearman’s test, *ρ*=−0.569, *p*-value = 0.000068). Bottom panel: SCG nucleotide divergence against genome divergence for the Tara haplotypes separated by MAG length. This gives the fractional divergence in SNVs between every pair of haplotypes (*I*) against the fractional divergence in 5% gene clusters across the whole genome (*C*). Data points are divided according to whether they derived from a MAG with genome length <1 Mbp. In a linear regression of genome divergence against nucleotide divergence, whether a MAG was <1 Mbp was a significant interaction (slope =0.11±0.02, *p*-value =5.95×10^−9^; slope interaction small = TRUE, 0.33±0.07, *p*-value = 3.51×10^−6^; overall adjusted R-squared = 0.6021, *p*-value =1.786×10^−12^). MAG metagenome-assembled genome, SCG single-copy core gene, SNV single-nucleotide variant

Having resolved variants on these 32 MAGs, we then applied the DESMAN haplotype deconvolution algorithm just as for the complex strain mock above, i.e. running all SNVs, varying the number of haplotypes *G*=1,…,7 and with the same heuristic strategy for determining the optimal haplotype number. The result was that all but three MAGs were predicted to possess strain variation with seventeen exhibiting two haplotypes, ten with three, and one each with four and five, respectively. The number of haplotypes inferred was highly significantly negatively correlated with MAG genome length (Spearman’s *p*-value =7.0×10^−4^; see Fig. 5 top panel).

#### Geographic patterns in Tara MAG haplotype abundance

**Fig. 6:**
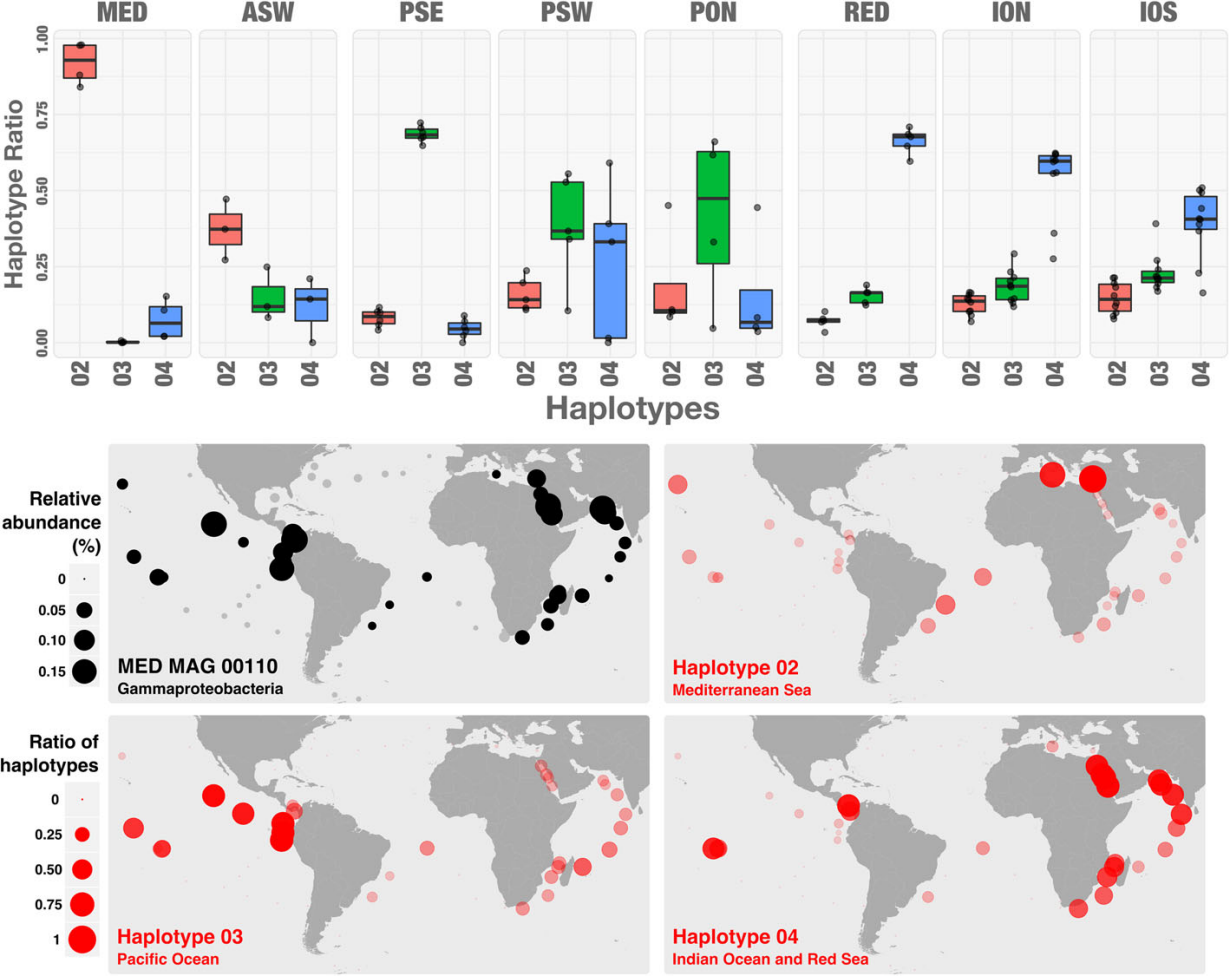
Geographic distribution of TARA_MED_MAG_00110 haplotypes. Top panel: Box plot of each haplotype’s relative abundance across the 11 regions where more than one sample had coverage greater than one. Bottom panel: The top left subpanel gives the total normalised relative abundance of the entire MAG. The other three subpanels give relative haplotype abundance for the three confidently inferred variants within this MAG. Results are shown for the 33 of 61 surface samples for which this MAG had coverage greater than 1. All three haplotypes were significantly associated with geographic region based on Kruskal–Wallis ANOVAs (H2: *χ* ^2^=20.9, *p*-value = 0.0074; H3: *χ* ^2^=17.8, *p*-value = 0.023; H4: *χ* ^2^=23.1, *p*-value = 0.0032). MAG metagenome-assembled genome, Mediterranean (MED), Athlantic South-West (ASW), Indian Ocean North (ION), Pacific South-East (PSE), Pacific South-West (PSW), Indian Ocean South (IOS), Pacific Ocean North (PON), Red Sea (RED)

In many cases, the haplotype relative abundance was observed to correlate with the spatial location of the plankton sample. An example for one MAG, a streamlined Gammaproteobacteria with an 0.89 Mbp genome, is shown in Fig. 6. Three strains were confidently inferred for this MAG and it can be seen that each strain is associated with a different geographical location. This was confirmed by performing ANOVA of each strain’s abundance against the discrete variable geographic region, corresponding to the 12 geographically co-located sample subsets (see Additional file 1: Table S9 and Additional file 1: Figure S11). For all three strains, the ANOVA was significant (Kruskal–Wallis: *p*-values = 0.0074, 0.023, 0.0032). These three strains differed by between 2.0% and 2.3% of the nucleotide positions on the SCGs. In fact, across all 73 inferred strains (from the 29 MAGs with haplotypes), we found that 42 or 57.5% exhibited a significant correlation with geographical region (Kruskal–Wallis *p*-value < 0.05).

#### Reconstruction of MAG accessory genomes

We next considered the entire pangenome, determining for each haplotype whether each gene in the MAG was present or absent and its sequence. This was done for each of the 29 MAGs with haplotypes. We then generated gene clusters from these inferred sequences for each MAG at 5% nucleotide difference and defined the genome divergence between each pair of haplotypes within a MAG as one minus the overlap in gene cluster complement between them (see ‘Methods’). There is a strong correlation between nucleotide divergence and whole genome divergence and the slope of this correlation was significantly larger for those MAGs with very small streamlined genomes (<1 Mbp) (interaction *p*-value = 3.51×10^−6^; see Fig. 5 bottom panel).

At present, there are insufficient isolate strains from marine organisms for us to validate the above phenomenon. However, we can at least check that the levels of genome divergence given SCG nucleotide divergence predicted from the metagenomes are consistent with known isolate strains. In Additional file 1: Figure S13, we show nucleotide divergence against genome divergence for three environmental organisms (*Methanosarcina mazei*, *Lactococcus lactis* and *Acinetobacter pittii*). This confirms that the results in Fig. 5 (bottom panel) are reasonable and that whilst core nucleotide divergence and genome divergence do correlate, there is a great deal of variation within species, and the relationship between the two varies from one species to another. In particular, in *Acinetobacter pittii* more genome divergence is observed for the same level of nucleotide divergence than for the other two species.

## Discussion

We have demonstrated for both *in silico* and real data sets the ability of DESMAN to infer and reconstruct microbial strains correctly from metagenomic data de novo using subtle nucleotide variations in mapping results.

Overall we did observe better results on the simple 20 genome mock, rather than the more realistic 210-genome *in silico* complex synthetic community but much of this is probably attributable to failures of the species binning and mapping algorithms rather than the haplotype inference per se. The most pertinent conclusion from the complex mock analysis is that just 36 universal SCGs are sufficient to resolve even closely related haplotypes for MAGs using as few as just ten SNVs. It is not necessary to use a larger collection of species-specific COGs as we did in the *E. coli* analyses. This is an important finding, as this strategy can be applied to all microbes, even those with no cultured isolates and, hence, no information on the pangenome. This was demonstrated by the Tara Oceans analysis. There we were able to elucidate biologically relevant patterns of strain diversification across a range of novel organisms, revealing geographical partitioning of strains and differences in relative rates of genome divergence with genome length. We discuss the biological implication of these results further below.

DESMAN was substantially more effective at reconstructing the five haplotypes of *E. coli* in our simple mock data set than Lineage [14]. The average SNV accuracy of the Lineage-predicted haplotypes was just 76.32% compared to 99.58% accuracy for DESMAN. For the more complex mock, the results were much closer between the two algorithms. There was an improvement associated with DESMAN but not dramatic. The reason for this is probably the difference in variant number. In the simple mock, 6,044 variants were identified for *E. coli* of which 1,000 were used for haplotype deconvolution. In contrast, in the complex mock using just the 36 SCGs, the most variants observed across the 25 MAGs was 303. The two haplotype inference algorithms are fundamentally similar despite Lineage being originally applied only after mapping to reference genomes. Lineage aims to exploit an additional level of information that is not used in DESMAN through the simultaneous construction of a phylogenetic tree between strains but DESMAN has a fully conjugate Gibbs sampler and a novel method based on NTF for initialisation. We hypothesise that these computational improvements give DESMAN an advantage on complex data sets, which may converge more slowly or be more sensitive to initial conditions but that on easier problems with smaller variant number, the inference accuracy is comparable. It would be worthwhile to extend DESMAN to include phylogenetic information, or conversely, introduce some of our improvements into the Lineage algorithm. This would further improve our collective ability to resolve complex pangenomes de novo from metagenomic assemblies. We were unable to run the ConStrains algorithm on our data, which in itself illustrates the advantage of a strategy in which we separate the steps of mapping, variant calling and haplotype inference. Although we suspect the partially heuristic and non-probabilistic approach utilised in ConStrains would have been unable to compete with the fully Bayesian algorithm employed in DESMAN.

The underlying haplotype inference model in DESMAN could be improved. Position-dependent error rates may be relevant given that particular sequence motifs are associated with high error rates on Illumina sequencers [26]. More fundamentally, we could develop models that do not assume independence across variant positions by combining information from the co-occurrence of variants in the same read with the modelling of strain abundances across multiple samples. This could be particularly relevant as single-molecule long read sequencers such as Nanopore become more commonly used [27]. In addition, it would have been preferable to have a more principled method for determining the number of strains present, rather than just examining the posterior mean deviance. This could be achieved through Bayesian non-parametrics, such as a Dirichlet process prior for the strain frequencies, allowing a potentially infinite number of strains to be present, with only a finite but flexible number actually observed [28]. Alternatively, a variational Bayesian approach could be utilised to obtain a lower bound on the marginal likelihood and this would be used to distinguish between models [29].

To the best of our knowledge, this is the first study to demonstrate that coverage across multiple samples can be used to infer gene counts across strains within a pangenome. We focussed on gene complement here but the underlying algorithm could be equally well applied to contigs just by calculating coverages and variants across a whole contig rather than on individual genes. We adopted the gene-centred approached because we can be confident that individual genes have been assembled correctly. This allowed us to resolve strain diversity and gene complement in entirely uncultured species. This revealed multiple biologically meaningful patterns across taxa within the Tara Oceans microbiome. We observed strain diversification in the vast majority of MAGs and the majority of these haplotypes (57.5%) were significantly correlated with geographic region, suggestive of local adaptation. The number of haplotypes in a MAG negatively correlated with metabolic complexity, indicating that the greatest strain diversity occurs in streamlined small genomes. This is not simply due to small genomes having lower GC content, since we observed that within a genome, non-variant positions were more likely to be AT. Instead, we believe that it reflects the importance of streamlining as a process for generating diversity in the plankton microbiome [30]. More intriguing is our observation that amongst highly streamlined organisms (genome length <1 Mbp), the rate of change in the overall genome, as measured by divergence in 5% gene clusters relative to changes in nucleotide difference on the core genes, is nearly three times greater than that for organisms with genomes greater than 1 Mbp. This suggests a difference in the way strain diversification and niche partitioning impacts the genome as genome length varies. For organisms with large genomes and complex metabolisms, niche differentiation requires less change in the genome with changes in gene expression level, for instance, driving ecological differences. For small genome organisms, strain generation and adaptation to new niches require larger genomic changes. This can be considered only a preliminary result that will require confirmation through isolate sequencing but it illustrates the power of DESMAN to generate novel hypotheses from metagenome data.

## Conclusion

We have demonstrated over a range of synthetic and real data sets that DESMAN is capable of accurate *de novo* resolution of strains from multi-sample metagenome data. We have also contributed to the growing realisation that intra-species strain diversity is endemic across many environments, underlining the need for such a tool.

The DESMAN pipeline is open-source software, and is available via the URL https://github.com/chrisquince/DESMAN.

## Methods

The DESMAN (De novo Extraction of Strains from MetAgeNomes) pipeline is a strategy for resolving both strain haplotypes and variations in gene content directly from short-read shotgun metagenome data. Our proposed approach comprises commonly employed steps of an assembly-based metagenomic binning workflow (such as co-assembly of data, annotation of resulting contigs, mapping short reads to the assembly and identification of genome bins), followed by preparing genome bins that match to the target organism for strain extraction using the novel DESMAN algorithm described below.

### Assembly and mapping

The first step is to co-assemble all reads from all samples. Chimeric contigs can confound the downstream analyses with DESMAN; therefore, the choice of assembler and the assembly parameters are important in targeting more accurate contigs rather than longer, but potentially chimeric ones, even if these selections result in relatively lower N50 values for the overall assembly. For our analyses, we used idba_ud [31], Ray [32] or MEGAHIT [33]. Assembly statistics are given in Additional file 1: Table S2. Note that the Tara Oceans assembly was not performed by us and the details are given in the original paper, although we do describe them briefly below [25].

Only contigs greater than 1 kbp in length were used for downstream analyses, and those greater than 20 kbp in length were fragmented into pieces smaller than 10 kbp [9]. The result of an assembly will be a set of *D* contigs with lengths in base pairs *L*_*d*_, and sequence composition *U*_*d*_ with elements *u*_*d*,*l*_ drawn from the set of nucleotides {A, C, G, T}.

Following co-assembly, we used bwa mem [34] to map raw reads in each sample individually back onto the assembled contigs. We then used samtools [35] and sequenza-utils [36] or bam-readcount to generate a four-dimensional tensor $\mathcal {N}$ reporting the observed base frequencies, *n*_*d*,*l*,*s*,*a*_, for each contig and base position in each sample *s* where *d*=1,…,*D*, *l*=1,…,*L*_*d*_, *s*=1,…,*S* and *a*=1,…,4, which represents an alphabetical ordering of bases 1→ A, 2→C, 3→G and 4→T.

Using this tensor, we calculated an additional *D*×*S* matrix, giving the mean coverage of each contig in each sample as:

$$x_{d,s} = \frac{n_{d,.,s,.}}{L_{d}}, $$

where we have used the convenient dot notation for summation, i.e. $ n_{d,.,s,.} \equiv \sum _{l = 1}^{L_{d}} \sum _{a = 1}^{4} n_{d,l,s,a} $.

### Contig clustering and target species identification

DESMAN can be used with any contig-binning method. We recommend using a clustering algorithm that takes both sequence composition and differential coverage of contigs into consideration. For the synthetic strain mock and the *E. coli* O104:H4 outbreak, we used the standard version of the CONCOCT algorithm [9]. For the complex strain mock, clustering was performed in two steps. Firstly, there is a standard CONCOCT run and then a re-clustering guided by SCG frequencies. This strategy has been released in the SpeedUp_Mp branch of the CONCOCT distribution https://github.com/BinPro/CONCOCT. The Tara binning strategy is described below and in the original study [25].

Irrespective of binning method, we assume that one or more of the resulting bins match to the target species and that they contain a total of *C* contigs with indices that are a subset of {1,…,*D*}. For convenience, we re-index the coverages and base frequency tensor such that *x*_*c*,*s*_ and *n*_*c*,*l*,*s*,*a*_ give the mean coverage and base frequencies in this subset, respectively.

### Identifying core genes in target species

The algorithm assumes a fixed number of strains in the target species. However, in general, not every gene in every contig will be present in all strains. We address this by identifying a subset of the sequences that occur in every strain as a single copy. Here we identify those core genes for *E. coli* by (1) downloading 62 complete *E. coli* genomes from the National Center for Biotechnology Information (NCBI) and (2) assigning COGs [37] to the genes in these genomes. COG identification was performed by RPS-BLAST for amino acid sequences against the NCBI COG database. This allowed us to identify 982 COGs that are both single copy and had an average of greater than 95% nucleotide identity between the 62 *E. coli* genomes. We denote these COGs as SCSGs.

We then identified SCSGs in MAGs that represent our target species, using RPS-BLAST, and created a subset of the variant tensor with base positions that fall within SCSG hits. We denote this subset as *n*_*h*,*l*,*s*,*a*_, where *h* is now indexed over the *H* SCSGs found and *l* is the position within each SCSG from 1,…,*L*_*h*_, which have lengths *L*_*h*_. We denote the coverages of these genes as *x*_*h*,*s*_.

For the *E. coli* analyses, we have reference genomes available and we could identify core genes, but this will not be the case in general for uncultured organisms, or even for those for which only a few isolates have been sequenced. In that case, we use a completely de novo approach, using 36 SCGs that are conserved across all species [9] but any other single-copy gene collection [38, 39] could serve the same purpose. We validated this strategy on the complex strain mock and then applied it to the Tara Oceans microbiome survey. The actual identification of SCGs and subsetting of variants proceeds as above. The result is a decrease in resolution, due to the decreased length of sequence that variants are called on, but as we demonstrate, it is still sufficient to resolve strains at low nucleotide divergence.

In real data sets, we have noticed that some core genes will, in some samples, have higher coverages than expected. We suspect that this is due to the recruitment of reads from low-abundance relatives that fail to be assembled. To account for this, we apply an additional filtering step to the core genes. All core genes should have the same coverage profile across samples. Therefore, we applied a robust filtering strategy based around the median absolute deviation [40]. We calculated the absolute divergence of each gene coverage from the median denoted $x^{m}_{s}$:

$$\operatorname{div}_{h,s} = |x_{h,s} - x^{m}_{s}|, $$

and then the median of these divergences, denoted by $\operatorname {div}^{m}_{s}$. If

$$\operatorname{div}_{h,s} > t \times \operatorname{div}^{m}_{s}, $$

we flag it as an outlier in that sample. Typically, we used *t*=2.5 as the outlier threshold. We only use genes that are not flagged in at least a fraction *f* of samples, where in these analyses *f* was set at 80%.

### Variant detection

Our algorithmic strategy begins with a rigorous method for identifying possible variant positions within the SCSGs. The main principle is to use a likelihood ratio test to distinguish between two hypotheses for each position. The null hypothesis $\mathcal {H}_{0}$ is that the observed bases are generated from a single true base under a multinomial distribution and an error matrix that is position-independent. We denote this error matrix *ε*, with elements *ε*_*a*,*b*_ giving the probability that a base *b* is observed when the true base is *a*. The alternative hypothesis $\mathcal {H}_{1}$, in which $\mathcal {H}_{0}$ is nested, is that two true bases are present. For this test, we ignore the distribution of variants over samples, working with the total frequency of each base across all samples:

$$t_{h,l,a} = n_{h,l,.,a}. $$

Although the generalisation of our approach to multiple samples would be quite straightforward, we chose not to do this for computational reasons and because we achieve sufficient variant detection accuracy with aggregate frequencies.

If we make the reasonable assumption that *ε*_*a*,*a*_>*ε*_*a*,*b*_ for *b*≠*a* for all *a*, then for a single true base with errors, the maximum likelihood solution for the true base is the consensus at that location, which we denote by the vector *M*_*h*_ for each SCSG with elements:

$$m_{h,l}^{0} = \arg \max_{a} \left(t_{h,l,a} \right). $$

The likelihood for $\mathcal {H}_{0}$ at each position is then the multinomial, assuming that bases are independently generated under the error model:

$$\mathcal{H}_{0}\left(t_{h,l,a} | \epsilon, r = m_{h,l}^{0} \right) = \prod_{a} \epsilon_{r,a}^{t_{h,l,a}} \frac{T_{h,l}!}{t_{h,l,a}!}, $$

where we use $r = m_{h,l}^{0}$ to index the maximum likelihood true base and *T*_*h*,*l*_ is the total number of bases at the focal position, *T*_*h*,*l*_=*t*_*h*,*l*,._. Similarly, for the two-base hypothesis, the maximum likelihood solution for the second base (or variant) is:

$$m_{h,l}^{1} = \arg \max_{a \not\in m_{h,l}^{0}} \left(t_{h,l,a} \right). $$

Then the likelihood for the hypothesis $\mathcal {H}_{1}$ at each position is

1 $$ \begin{aligned} &\log \mathcal{H}_{1}\left(t_{h,l,a} | \epsilon, r = m_{h,l}^{0}, s = m_{h,l}^{1}, p_{h,l} = p \right)\\ &\quad= \prod_{a} (p \epsilon_{r,a} + (1 - p) \epsilon_{s,a})^{tn}\frac{T_{h,l}!}{t_{h,l,a}!}, \end{aligned}   $$

where we have introduced a new parameter for the relative frequency of the consensus base, *p*. We set an upper bound on this frequency, *p*_max_, such that *p*_*l*_=1−*p*_max_ corresponds to the minimum observable variant frequency. For the synthetic mock community, we set *p*_*l*_=0.01, i.e. 1%. For the other two real data sets, where we want to be more conservative, we used *p*_*l*_=0.03. For each position, we determine this by maximum likelihood by performing a simple one-dimensional optimisation of Eq. 1 with respect to *p*. Having defined these likelihoods, our ratio test is:

2 $$ -2\log{\frac{\mathcal{H}_{0}}{\mathcal{H}_{1}}},   $$

which will be approximately distributed as a chi-squared distribution with one degree of freedom. Hence, we can use this test to determine *p*-values for the hypothesis that a variant is present at a particular position.

There still remains the question of how to determine the error matrix, *ε*. We assume that these errors are position-independent, and to determine them, we adopt an iterative approach resembling expectation maximisation. We start with a rough approximation to *ε*, categorise positions as variants or not, and then recalculate *ε* as the observed base transition frequency across all non-variant positions. We then re-classify positions and repeat until *ε* and the number of variants detected converge. Finally, we apply a Benjamini–Hochberg correction to account for multiple testing to give a FDR or *q*-value for a variant at each position [41]. The variant positions identified by this procedure should represent sites where we are confident variation exists in the MAG population at greater than 1% frequency. However, we cannot be certain that this variation is necessarily from the target species because of potential recruitment of reads from other organisms; therefore, we prefer the term single-nucleotide variants (SNVs) for these positions, rather than single-nucleotide polymorphisms (SNPs), which we keep for variant positions in isolate genomes.

### Probabilistic model for variant frequencies

Having identified a subset of positions that are likely variants, the next step of the pipeline is to use the frequencies of those variants across multiple samples to link the variants into haplotypes. We use a fairly low *q*-value cut-off for variant detection, using all those with FDR <1.0×10^−3^. This ensures that we limit the positions used in this computationally costly next step to those most likely to be true variants. The cost is that we may miss some low-frequency haplotypes but these are unlikely to be confidently determined anyway. We will index the variant positions on the SCSGs by *v* and for convenience keep the same index across SCSGs, which we order by their COG number, so that *v* runs from $1,\ldots, N_{1},\ldots, N_{1} + N_{2},\ldots,\sum _{h} N_{h}$, where *N*_*h*_ is the number of variants on the *h*th SCSG and keep a note of the mapping back to the original position and SCSG denoted *v*→(*l*_*v*_,*h*_*v*_). We denote the total number of variants by $V = \sum _{h} N_{h}$ and the tensor of variant frequencies obtained by subsetting $n_{h_{v},l_{v},s,a} \rightarrow n_{v,s,a}$ on the variant positions as $\mathcal {N}$.

#### Model likelihood

The central assumption behind the model is that these variant frequencies can be generated from *G* underlying haplotypes with relative frequencies in each sample *s* denoted by *π*_*g*,*s*_, so that *π*_.,*s*_=1. Each haplotype then has a defined base at each variant position denoted *τ*_*v*,*g*,*a*_. To encode the bases, we use four-dimensional vectors with elements ∈{0,1}, where a 1 indicates the base and all other entries are 0. The mapping to bases is irrelevant but we use the same alphabetical ordering as above, thus *τ*_*v*,*g*,._=1.

We also assume a position-independent base transition or error matrix giving the probability of observing a base *b* given a true base *a* as above, *ε*_*a*,*b*_. Then, assuming independence across variant positions, i.e. explicitly ignoring any read linkage, and more reasonably between samples, the model likelihood is a product of multinomials:

3 $$ \begin{aligned} \mathcal{L}\left(\mathcal{N} | \pi, \tau,\epsilon \right) &= \prod_{v=1}^{V} \prod_{s = 1}^{S} \prod_{a = 1}^{4} \left(\sum_{b=1}^{4} \sum_{g = 1}^{G} \tau_{v,g,b} \pi_{g,s} \epsilon_{b,a} \right)^{n_{v,s,a}}\\ &\quad\times\frac{n_{v,s,.}!}{n_{v,s,a}!}. \end{aligned}   $$

#### Model priors

Having defined the likelihood, here we specify some simple conjugate priors for the model parameters. For the frequencies in each sample, we assume symmetric Dirichlet priors with parameter *α*:

$$P(\pi | \alpha) = \prod_{s} \text{Dir} (\pi_{g,s} | \alpha). $$

Similarly, for each row of the base transition matrix, we assume independent Dirichlets:

$$P(\epsilon | \delta) = \prod_{a} \text{Dir} (\epsilon_{a,b} | \delta) $$

with parameter *δ*. Finally, for the haplotypes themselves (*τ*), we assume independence across positions and haplotypes, with uniform priors over the four states:

$$P(\tau_{v,g,a}) = \frac{1}{4}. $$

#### Gibbs sampling strategy

We will adopt a Bayesian approach to inference of the model parameters, generating samples from the joint posterior distribution:

4 $$ P(\tau, \pi, \epsilon | \mathcal{N}) = \frac{P(\tau, \pi, \epsilon,\mathcal{N})}{P(\mathcal{N})}.   $$

We use a Gibbs sampling algorithm to sample from the conditional posterior of each parameter in turn, which will converge on the joint posterior given sufficient iterations [42]. The following three steps define one iteration of the Gibbs sampler:

1. The conditional posterior distribution for the haplotypes, *τ*_*v*,*g*,*a*_, is
  $$P(\tau | \epsilon, \pi, \mathcal{N}) \propto P(\mathcal{N} | \tau, \pi, \epsilon) P(\tau). $$
  Each variant position contributes independently to this term, so we can sample each position independently. The haplotype assignments are discrete states, so their conditional will also be a discrete distribution. We sample *τ* for each MAG in turn, from the conditional distribution for that genome, with the assignments of the other genomes fixed to their current values:
  5 $$ \begin{aligned} P\left(\tau_{v,g,a}|\pi, \epsilon, \mathcal{N},\tau_{v,h \neq g,a}\right) \propto \prod_{s} \prod_{a} \left(\sum_{g} \sum_{b} \tau_{v,g,b} \pi_{g,s} \epsilon_{b,a} \right)^{n_{v,s,a}}. \end{aligned}   $$
2. To sample *ε*, we introduce an auxiliary variable, *ν*_*v*,*s*,*a*,*b*_, which gives the number of bases of type *a* that were generated by a base of type *b* at location *v* in sample *s*. Its distribution, conditional on *τ*, *π*, *ε* and $\mathcal {N}$, will be multinomial:
  $$P\left(\nu_{v,s,a,b} | \tau, \pi, \epsilon, \mathcal{N}\right) = \prod_{b = 1}^{4} \left(\frac{\zeta_{v,s,a,b}^{\nu_{v,s,a,b}} }{\nu_{v,s,a,b}!}\right)n_{v,s,a}!, $$
  where
  $$\zeta_{v,s,a,b} = \frac{\sum_{g} \tau_{v,g,b} \pi_{g,s} \epsilon_{b,a}}{\sum_{a} \sum_{g} \tau_{v,g,b} \pi_{g,s} \epsilon_{b,a}}. $$
  Since the multinomial is conjugate to the Dirichlet prior assumed for *ε*, then we can easily sample *ε* conditional on *ν*:
  $$P(\epsilon_{b,a} | \delta, \nu) = \text{Dir} (\nu_{.,.,a,b} + \delta). $$
3. To sample *π*, we define a second auxiliary variable *ξ*_*v*,*s*,*a*,*b*,*g*_, which gives the number of bases of type *a* that were generated by a base of type *b* at each position *v* from haplotype *g* in sample *s*. This variable conditioned on *τ*, *π*, *ε* and *ν* will be distributed as:
  $$P(\xi_{v,s,a,b,g} | \tau, \pi, \epsilon, \nu) = \prod_{g} \left(\frac{\psi_{v,s,a,b,g}^{\xi_{v,s,a,b,g}}}{\xi_{v,s,a,b,g}!}\right) \nu_{v,s,a,b}! $$
  with
  $$\psi_{v,s,a,b,g} = \frac{\tau_{v,g,b} \pi_{g,s} \epsilon_{b,a}}{\sum_{g} \tau_{v,g,b} \pi_{g,s} \epsilon_{b,a}}. $$
  Similarly, *π* is also a Dirichlet conditional on *ξ*:
  $$P(\pi_{g,s} |\xi_{.,s,.,.,g}) = \text{Dir}\left(\xi_{.,s,.,.,g} + \alpha \right). $$

#### Initialisation of the Gibbs sampler

Gibbs samplers can be sensitive to initial conditions. To ensure rapid convergence on a region of high posterior probability, we consider a simplified version of the problem. We calculate the proportions of each variant at each position in each sample:

$$p_{v,s,a} = \frac{n_{v,s,a}}{n_{v,s,.}}. $$

Then an approximate solution for *τ* and *π* will minimise the difference between these observations, and

$$\hat p_{v,s,a} = \sum_{g} \tau_{v,g,a} \pi_{g,s}. $$

If we relax the demand that *τ*_*v*,*g*,*a*_∈0,1 and instead allow it to be continuous, then solving this problem is an example of an NTF, which itself is a generalisation of the better known NMF problem [19]. We adapted the standard multiplicative update NTF algorithm that minimises the generalised Kullback-Leibler divergence between *p* and $\hat p$:

$$\mathrm{D}_{KL} (p | \hat p) = \sum_{v} \sum_{s} \sum_{a} p_{v,s,a} \log \left(\frac{p_{v,s,a}}{\hat p_{v,s,a}} \right) + \hat p_{v,s,a} - p_{v,s,a}. $$

This is equivalent to assuming that the observed proportions are a sum of independent Poisson-distributed components from each haplotype, ignoring the issue that the Poisson is a discrete distribution [43]. The standard multiplicative NMF algorithm can be applied to our problem [44] by rearranging the *τ* tensor as a 4*V*×*G* matrix *τ* *w*,*g*′≡*τ*_*v*,*g*,*a*_, where *w*=*v*+(*a*−1)*V*. By doing so, we have created a matrix from the tensor by stacking each of the base components of all the haplotypes vertically. Similarly, we rearrange the variant tensor into a 4*V*×*S* matrix with elements $n^{\prime }_{w,s} \equiv n_{v,s,a}$, where *w*=*v*+(*a*−1)*V*. The update algorithms become:

$$\begin{array}{@{}rcl@{}} \tau{\prime}_{w,g} & \leftarrow & \tau{\prime}_{w,g} \frac{\sum_{s} \pi_{g,s} n{\prime}_{w,s}/(\tau{\prime}.\pi)_{w,s}}{\sum_{s} \pi_{g,s}},\\ \pi_{g,s} & \leftarrow & \pi_{g,s} \frac{\sum_{w} \tau{\prime}_{w,g} n{\prime}_{w,s}/(\tau{\prime}.\pi)_{w,s}}{\sum_{w} \tau{\prime}_{w,g}}. \end{array} $$

Then we add a normalisation step:

$$\begin{array}{@{}rcl@{}} \tau{\prime}_{w,g} & = & \tau{\prime}_{w,g}/\sum_{a} \tau{\prime}_{v + (a -1).V,g},\\ \pi_{g,s} & = & \pi_{g,s}/\sum_{g} \pi_{g,s}. \end{array} $$

Having run the NTF until the reduction in D_*KL*_ was smaller than 10^−5^, we discretised the predicted *τ* values such that the predicted base at each position for each haplotype was the one with the largest *τ* ^′^. We used these values with *π* as the starting point for the Gibbs sampler.

#### Implementation of the Gibbs sampler

In practice, following initialisation with the NTF, we run the Gibbs sampling algorithm twice for a fixed number of iterations. The first run is a burn-in phase to ensure convergence, which can be checked via manual inspection of the time series of parameter values. The second run is the actual sampler, from which *T* samples are stored as samples from the posterior distribution, *θ*_*t*_=(*τ*_*t*_,*π*_*t*_,*ε*_*t*_) with *t*=1,…,*T*. These can then be summarised by the posterior means, $\hat {\theta } = \sum _{t} \theta _{t} /T$, and used in subsequent downstream analysis. We also store the sample with the maximum log-posterior, denoted *θ* ^∗^=(*τ* ^∗^,*π* ^∗^,*ε* ^∗^), if a single most probable sample is required. For many data sets, *V* will be too large for samples to be generated within a reasonable time. Fortunately, we do not need to use all variant positions to calculate *π* with sufficient accuracy. We randomly selected a subset of the variants, ran the sampler, obtained samples (*π*_*t*_,*ε*_*t*_) and use these to assign haplotypes to all positions, by running the Gibbs sampler just updating *τ* sequentially using Eq. 5 and iterating through the stored (*π*_*t*_,*ε*_*t*_).

#### Determining the number of haplotypes and haplotype validation

Ideally the Bayes factor or the model evidence, the denominator in Eq. 4, would be used to compare between models with different numbers of haplotypes. Unfortunately, there is no simple reliable procedure for accurately determining the Bayes factor from Gibbs sampling output. For this reason, we suggest examining the posterior mean deviance [45]:

$$ D = \frac{\sum_{t} -2 \log\left[\mathcal{L}\left(\mathcal{N} | \pi_{t}, \tau_{t},\epsilon_{t} \right)\right]}{T}. $$

As the number of haplotypes increases, the model will fit better and *D* will decrease. When the rate of decrease is sufficiently small, then we conclude that we have determined the major abundant haplotypes or strains present. This method is ambiguous but has the virtue of not making any unwarranted assumptions necessary for approximate estimation of the Bayes factor. To validate individual haplotypes, we compare replicate runs of the model. Since the model is stochastic, then different sets of haplotypes will be generated each time. If in replicate runs we observe the same haplotypes, then we can be confident in their validity. Therefore, calculating the closest matching haplotypes across replicates gives an estimate of our confidence in them. We define the mean SNV uncertainty for a haplotype as the fraction of positions for which it differs from its closest match in a replicate run, averaged over all the other replicates.

For predictions, the run used was the one with lowest posterior mean deviance giving the predicted *G*. Parameter predictions were taken as the posterior mean over the sampled values. For the haplotype sequences, these means were discretised by setting *τ*_*v*,*g*,*m*_=1 and *τ*_*v*,*g*,*a*≠*m*_=0 where *m*= arg max*a* *τ*_*v*,*g*,*a*_.

When analysing multiple clusters, an automatic method of inferring the true number of haplotypes is required. To provide this, we developed a heuristic algorithm like the human-guided strategy discussed above. As *G* increases, the mean posterior deviance must decrease but when the rate of decrease is sufficiently small, then we can conclude that we have determined the major abundant haplotypes present. We, therefore, ran multiple replicates (typically five) of the haplotype resolution algorithm for increasing *G*=1,…,*G*_max_, and set a cut-off *d* (set at 5% for the studies presented here). When the successive reduction in posterior mean deviance averaged over replicates fell below this value, i.e. $({\mathbb E}[D_{G-1}] - {\mathbb E}[D_{G}])/ {\mathbb E}[D_{G-1}] < d$, we used *G*_U_=*G*−1 as an upper limit on the possible number of resolved haplotypes. We considered all *G* between 1 and *G*_U_ and at each value of *G*, we calculated the number of haplotypes that had a mean SNV uncertainty (see above) below 10% and a mean relative abundance above 5%. We chose the optimal *G* to be the one that returned the most haplotypes satisfying these conditions of reproducibility and abundance.

### Resolving the accessory genome

Having resolved the number of strains and their haplotypes on the core genome, we now consider the question of how to determine the accessory genome for each strain. The strategy below could equally well be applied to either contigs or genes called on those contigs. In our experience, contigs are frequently chimeric, and we have achieved better results with gene-based approaches. If contig assignments are required, then a simple consensus of the genes on a contig can be used. We will, therefore, describe a gene-based analysis keeping in mind that contigs could be used interchangeably.

We should have already assigned genes on all contigs in the target bin or bins above. Now we consider not just the SCSGs but all genes, which we will index *f*=1,…,*F*. Just as for the SCSGs, we can identify variant positions on the total gene set using Eq. 2. In fact, we apply a slightly modified version of this strategy in this case because of the large number of positions to be screened, replacing the one-dimensional optimisation of *p* with an estimation of the frequency of the consensus base as the ratio of the observed number of consensus bases to the total, $p = t_{h,l,m_{h,l}^{0}} / T_{h,l}$.

We will denote the number of variant positions associated with gene *f* by *N*_*f*_. In this case, we do need to keep track of which variant maps to which gene explicitly, so we will consider a four-dimensional variant tensor denoted $\mathcal {M}$ with elements *m*_*f*,*l*,*s*,*a*_ where *l* is indexed from 1,…,*N*_*f*_. This is generated by subsetting the original contig variant tensor $\mathcal {N}$ to the variants associated with each gene. In practice, to speed up the algorithm we use only a random subset of variants (20 was used here), since all variants contain the information necessary to determine which gene is present in which strain. An additional source of information that we will use is the average coverage of each gene across samples. This is the exact analogue of the contig coverage introduced above and we will denote it with the same symbol, i.e. $\mathcal {X}$ with elements *x*_*f*,*s*_.

Determining the accessory genome corresponds to inferring the copy number of each gene in each strain. We denote this integer as *η*_*f*,*g*_, for each of the genes *f*=1,…,*F* associated with the species in each strain, *g*=1,…,*G*. The ideas we present here could be extended to multi-copy genes; however, the current implementation of DESMAN assumes that all genes are present in zero or one copies, *η*_*f*,*g*_∈{0,1}. This simplifies the implementation considerably and in real assemblies the vast majority of genes are either present or absent in a strain. For example, for the STEC genome, this is true of 98.8% of the genes.

The first step is to determine the likelihood. We assume that this is separable for the variants and the coverages. This is an approximation, as the variant positions will contribute to the mean coverage calculation. Formally, we assume:

$$\mathcal{L}\left(\mathcal{M}, \mathcal{X}| \eta, \pi, \tau,\epsilon \right) = \mathcal{L}^{v}\left(\mathcal{M}| \eta, \pi, \tau,\epsilon \right).\mathcal{L}^{x}\left(\mathcal{X}| \eta, \gamma \right). $$

The first likelihood is, like Eq. 3, a product of multinomials:

6 $$ \begin{aligned} \mathcal{L}^{v}\left(\mathcal{M} | \eta, \pi, \tau,\epsilon \right) &= \prod_{f=1}^{F} \prod_{l=1}^{N_{f}} \prod_{s = 1}^{S} \prod_{a = 1}^{4} \left(\sum_{b=1}^{4} \sum_{g \in G_{f}} \tau_{f,l,g,b} \pi{\prime}^{f}_{g,s} \epsilon_{b,a} \right)^{m_{f,l,s,a}}\\ &\quad\times\frac{m_{f,l,s,.}!}{n_{f,l,s,a}!}. \end{aligned}   $$

The difference is that now the sum over the strains *g* are only those for which *η*_*f*,*g*_>0, those which actually possess a copy of gene *f*, a set that we denote *g*∈*G*_*f*_. The relative frequencies then have to be renormalised accordingly so that:

$$ \pi{\prime}^{f}_{g,s} = \frac{\pi_{g,s}}{\sum_{g \in G_{f}} \pi_{g,s}}. $$

The likelihood for the coverages is somewhat simpler. We know the relative proportions of each strain in each sample, *π*_*g*,*s*_. We also know the mean total coverage on the core genes:

$$Y_{s} = n_{.,.,s,.}/\sum_{l=1}^{H} L_{h}. $$

Therefore, we can calculate the coverage associated with each strain:

$$\gamma_{g,s} = \pi_{g,s} Y_{s}. $$

We can make the approximation that each copy of a contig from a strain contributes independently to the total mean coverage observed for that contig in a particular sample. If we further assume that this contribution is Poisson distributed with mean *γ*_*g*,*s*_, then the total contribution will be from the superposition property of Poisson distributions, which are again Poisson with mean $\lambda _{f,s} = \sum _{g} \eta _{f,g} \gamma _{g,s}$. Thus,

7 $$ \mathcal{L}^{x}\left(\mathcal{X}| \eta, \gamma \right) = \prod_{f=1}^{F} \prod_{s = 1}^{S} \exp\left(-\lambda_{f,s}\right) \lambda_{f,s}^{x_{f,s}} \frac{1}{\Gamma\left(x_{f,s} + 1\right)}.   $$

Our strategy for sampling the gene assignments *η*_*f*,*g*_ is to keep the relative proportions of each strain in each sample, *π*_*g*,*s*_, and the error matrix, *ε*_*b*,*a*_, fixed at their posterior mean values $(\hat {\pi },\hat {\epsilon })$. We then use a Gibbs sampler to jointly sample both the *η*_*f*,*g*_ and the haplotypes of those strains *τ*_*f*,*l*,*g*,*a*_. In general, we assume a geometric prior for the *η*_*f*,*g*_, so that $P(\eta _{f,g} = \eta) = \eta _{s}^{\eta }/Z$, where *η*_*s*_ is less than 1 to penalise multi-copy genes, although here, as mentioned above, we restrict ourselves to binary *η*, and *Z* is a normalisation constant. Each gene contributes to the likelihood independently and so can be sampled independently. We can, therefore, loop through the genes, sampling *η* for each strain conditional on the other genomes fixed at their current values:

8 $$ \begin{aligned} P(\eta_{f,g} = \eta ; \tau_{f,l,g,a} = \tau_{l,a} | \hat{\pi},\hat{\epsilon},\tau_{f,h \neq g,a},\eta_{f,h \neq g}) \propto \\ \mathcal{L}^{v}\left(\mathcal{M}| \eta, \hat{\pi}, \tau,\hat{\epsilon} \right). \mathcal{L}^{x}\left(\mathcal{X}| \eta, \hat{\gamma}, \right) P(\eta)P(\tau),  \end{aligned}  $$

substituting Eqs. 6 and 7 into this and using uniform priors for *τ*.

To improve the speed of convergence of this sampler, we developed an approximate strategy to initialise *η*_*f*,*g*_ using just the coverages, *x*_*f*,*s*_. If we ignore for now that *η*_*f*,*g*_ is discrete, then the maximum likelihood prediction for *η*_*f*,*g*_ from Eq. 7 will correspond to minimising the generalised Kullback-Leilber divergence between the observed coverages *x*_*f*,*s*_, and their predictions, $\hat x_{f,s} = \sum _{g} \eta _{f,g} \gamma _{g,s}$:

$$\mathrm{D}_{KL} \left(x_{f,s} | \hat x_{f,s}\right) = \sum_{c} \sum_{s} x_{f,s} \log \left(\frac{x_{f,s}}{\hat x_{f,s}} \right) + \hat x_{f,s} - \hat x_{f,s}. $$

This also corresponds to NMF but with a fixed estimate for *γ*_*g*,*s*_. Therefore, to solve it for *η*_*f*,*g*_, we need only one of the multiplicative update rules [44]:

9 $$ \eta_{f,g} \leftarrow \eta_{f,g} \frac{\sum_{s} \gamma_{g,s} x_{f,s}/(\eta.\gamma)_{f,s}}{\sum_{s} \gamma_{g,s}},   $$

which gives continuous estimates for *η*_*f*,*g*_, but we round these to the nearest integer for discrete copy number predictions.

The sampler is initialised using Eq. 9 before applying a burn-in and sampling phase using Eq. 8. Typically, we have found that a relatively small number of samples, just 20, is sufficient before the *η* values converge. We also use only a random subset of the variant positions (again 20) for the *η* sampling as discussed above. Optionally, we then allow an additional sampling phase to determine the remaining *τ*, the haplotype sequences, with the *η* values fixed at their posterior mean values, if required.

#### Calculating genome divergence

To determine a measure of overall genome divergence that takes into account both which genes are present in a genome and how divergent in nucleotide sequence those genes are, we calculated for each strain both the gene complement, *η*_*f*,*g*_, and the gene haplotype, *τ*_*f*,*l*,*g*,*a*_. We converted the haplotypes into gene sequences using the contig references, and clustered all the sequences from all strains in a MAG at 5% nucleotide identity using the clustering algorithm of vsearch [46] and for each strain mapped its gene sequences back onto these cluster centroids and assigned each strain gene sequence to its closing matching cluster. Each strain is then represented as a vector of 5% gene cluster frequencies *υ*_*g*,*c*_ where *c* indexes the gene clusters, of which we assume there are *C* in total. A measure of genome divergence between two strains *g* and *h* is then:

10 $$ d_{g,h} = 1 - \frac{\sum_{c} \min(\upsilon_{g,c}, \upsilon_{h,c})}{\sum_{c} \max(\upsilon_{g,c}, \upsilon_{h,c})}.  $$

Using this measure, the divergence is 0 if two strains contain all the same genes and their sequences are within 5% nucleotide identity of each other. Conversely, the divergence is 1 if they share no gene sequences within 5% identity.

### Creation of complex strain mock

We simulated a complex community comprising 100 different species and 210 strains. The exact strains used are detailed in Additional file 3. The 100 species were chosen randomly from bacteria and archaea for which multiple complete genomes were available from the NCBI. They span a wide range of taxonomic diversity deriving from 10 separate phyla, 49 families and 74 genera, although with an inevitable bias to Proteobacteria. For each species, between one and five separate genomes were used in the simulation with a species strain frequency distribution of (1:50,2:20,3:10,4:10,5:10), i.e. there were 50 species with no strain variation and ten comprised five strains.

We simulated reads from these genomes using the ART sequence simulator [47], run through a set of custom scripts, which are collated in the repository https://github.com/chrisquince/StrainMetaSim. In total, 96 samples were generated, each comprising approximately 6.25 million 2×150 bp paired-end reads with an average insert length of 300 bp with a standard Illumina error profile. This approximates to running the 96 samples on one run of a HiSeq2500 in rapid run mode using dual flow cells (assuming 180 Gbp per run).

We modelled the species abundances across samples using normalised log-normal distributions. We assumed each species, indexed *t*=1,…,*T*, to have a mean and standard deviation log abundance of *μ*_*t*_ and *σ*_*t*_, respectively, such that its relative frequency *n*_*t*,*s*_ in sample *s* is generated by:

11 $$ y_{t,s} \sim \mathcal{N}(\mu_{t}, \sigma_{t})  $$

and then

12 $$ n_{t,s} = \frac{\mathrm{e}^{y_{t,s}}}{\sum_{t} \mathrm{e}^{y_{t,s}}}.  $$

The log-normal parameters, *μ*_*t*_ and *σ*_*t*_, for each species are themselves generated from a normal (mean = 1.0, standard deviation = 0.25) and gamma distribution (shape = 1.0, scale = 1.0), respectively. Then, within each species, we used a symmetric Dirichlet distribution to model the relative strain frequencies:

13 $$ \rho_{s} \sim \text{Dir} (\mathbf{a}),  $$

where the vector **a** has a dimensionality equal to the number of strains in that species. In practice, we used a unit vector for this parameter. The relative frequency for each strain *d* is then:

14 $$ \kappa_{d \in t,s} = n_{t,s}\rho_{s,d}.  $$

This gives the probability that a read in a given sample derives from a given strain. The strain coverage is then

15 $$ z_{d,s} = \frac{R \kappa_{d,s} N_{s}}{L_{d}},   $$

where *N*_*s*_ is the number of reads in sample *s*, *R* is the read length and *L*_*d*_ is the strain genome length. The program ART was used to generate simulated reads with this level of coverage from each strain genome in each sample. The result was that the number of reads varied slightly, since reads are discrete and coverage a continuous quantity. In total, 599,067,690 paired reads were generated. These reads were then collated into samples to simulate the community.

#### Assignment of contigs to species and genes to strains

To determine which contig derived from which species, we considered the reads that mapped onto it. Each read has a known genome assignment that derives from the sequence simulator. We, therefore, assign a contig to the species that the majority of its reads derive from. There were relatively few chimeric contigs of the 74,581 contig fragments greater than 1,000 bp in length. Only 228 (0.3%) had less than 90% of mapped reads deriving from the assigned species. Similarly, for each individual gene called across all contigs by prodigal, we determined the fraction of reads deriving from each strain genome for comparison with the inferred gene assignments from the second step of the DESMAN pipeline.

### Tara Oceans MAG collection

The details of the generation of the 957 non-redundant Tara Oceans MAGs are given in the original manuscript [25]. Briefly, the 93 Tara Oceans metagenome samples (30.9 billion filtered reads) from the planktonic size fraction (61 surface samples and 32 samples from the deep chlorophyll maximum layer) were grouped into 12 metagenomic sets based on geographic location. These geographic locations are detailed in Additional file 1: Table S9 and Figure S11. Each set was then independently co-assembled using MEGAHIT [33] and all contigs >2.5 kbp (>5 kbp for the Southern Ocean) were binned using an initial automatic binning with CONCOCT, followed by interactive refinement with the Anvi’o interface as described in [10]. Redundant MAGs, i.e. the same genome appearing from multiple co-assemblies, were identified using a combination of average nucleotide identity (>99%) on at least 75% shared genome and relative abundance correlation (Pearson’s correlation >0.9). CheckM was used to infer the taxonomy of MAGs based on the proximity of 43 single-copy gene markers within a reference genomic tree [48]. For each MAG, genes were called using the program prodigal with the -p meta metagenomics flag [49]. The genes were annotated to KEGG orthologues by amino acid alignments against KEGG FTP Release 2014-04-14 using RAPSearch2. A KEGG pathway module was considered present in a MAG if at least 75% of the orthologues of at least one pathway through that module were found. The genes were also annotated to COGs [37] and the fraction of the 36 single-copy core COGs (SCGs) identified in Alneberg et al. [9] that were found in a single copy were used to determine MAG purity and completeness.

## Additional files

Additional file 1 Containing 10 supplementary tables and 13 supplementary figures. (PDF 1096 kb)

Additional file 2 Separate text file containing variant prediction results in the complex mock. (TSV 4 kb)

Additional file 3 Separate text file containing strains used in the complex mock. (TSV 12 kb)
