# Supplementary material for: DESMAN: a new tool for de novo extraction of strains from metagenomes
Source: Genome Biol. 2017 Sep 21;18:181. doi: 10.1186/s13059-017-1309-9 (PMC5607848; doi:10.1186/s13059-017-1309-9)
Supplement: Supplementary file 1 — Containing 10 supplementary tables and 13 supplementary figures. (PDF 1096 kb) [file 13059_2017_1309_MOESM1_ESM.pdf]

Supplementary Materials: DESMAN: a new tool for de novo extraction of strains from metagenomes

Identifier	Species	NCBI Acc_No
ESC_COL_K12	Escherichia coli K-12	NC_000913.3
ESC_COL_O104	Escherichia coli STEC outbreak	NC_018658.1, NC_018659.1, NC_018660.1, NC_018666.1
ESC_COL_O157	Escherichia coli Sakai	NC_002127.1, NC_002128.1, NC_002695.1
ESC_COL_UT189	Escherichia coli UPEC	NC_007941.1, NC_007946.1
ESC_COL_O127	Escherichia coli EPEC	NC_011601.1, NC_011602.1, NC_011603.1
BAC_VUL	Bacteroides vulgatus ATCC 8482	NC_009614.1
BAC_THE	Bacteroides thetaiotaomicron VPI-5482	NC_004703.1
BAC_FRA	Bacteroides fragilis 638R	NC_016776.1
BAC_HEL	Bacteroides helcogenes P 36-108	NC_014933.1
BAC_SAL	Bacteroides salanitronis DSM 18170	NC_015166.1
CLO_DIF	Clostridium difficile BI1	NC_017179.1
FAE_PRA	Faecalibacterium prausnitzii L2-6	NC_021042.1
ROS_HOM	Roseburia hominis A2-183	NC_015977.1
RUM_ALB	Ruminococcus albus 7	NC_014833.1
EUB_REC	Eubacterium rectale ATCC 33656	NC_012781.1
BIF_LON	Bifidobacterium longum subsp. longum BBMN68	NC_014656.1
ENT_FAE	Enterococcus faecalis D32	NC_018223.1
AKK_MUC	Akkermansia muciniphila ATCC BAA-835	NC_010655.1
LAC_BRE	Lactobacillus brevis ATCC 367	NC_008499.1
HEL_PYL	Helicobacter pylori Rif1	NC_018937.1

Table S1: The 20 genomes used in the strain sythetic mock community.

Data set	No. samples	No. reads	Total length	No. contigs > 1kbp	No. contigs > 2kbp	N50 length
‘Strain’ mock	64	1.504×10^9	60Mbp	7,545	6,002	10,000
‘Complex strain’ mock	96	1.198×10^9	409 Mbp	74,580	51,406	10,000
E. coli O104:H4 outbreak	53	2.2×10^8	317 Mbp	142,723	47,918	2,402

Table S2: Co-assembly statistics for three of the data sets used in this study. For the Tara assembly statistics please see the original paper [1]. The *E. coli* O104:H4 outbreak was assembled using `Ray` with kmer size 41 [2]. The synthetic ‘strain’ mock was assembled using `idba_ud` with default parameters and kmer sizes increasing from 21 to 121 and the ‘pre_correction’ flag. The ‘complex strain’ mock was assembled with `MEGAHIT` and default parameters [3]. Contigs greater than 20kbp in length were fragmented into 10kbp pieces. The N50 length was calculated for contigs greater than 1kbp.

Table S3: Confusion matrix for variant detection in the ‘strain’ mock.

	Actual	
Predicted	False	True
False	249,584	125
True	6	6,038

A contingency table or confusion matrix giving the frequency of positions that were (rows - True) or were not (rows - False) predicted to be variants with a FDR or q-value < 1.0e-3 that actually were (columns - True) or were not (columns - False). Overall prediction accuracy was 99.9% with a precision of 99.9% and a recall of 97.9%.

Table S4: Comparison of inferred strains to true reference genomes in the 'strain' mock.

	Strain genomes				
Predicted	EC_O127	EC_O104	EC_O157	EC_K12	EC_UT189
H_0	3532	1606	2083	16	3603
H_1	3728	2306	39	2084	3753
H_2	1715	3681	3755	3587	35
H_3	10	3637	3718	3522	1728
H_4	3628	27	2288	1591	3681

This table compares the predicted strains for one run of the algorithm with the five known *E. coli* genomes at each of the 6,044 variant positions. The integer frequencies give the number of positions where each strain (row) differs from a genome (column). The overall accuracy rate for correct prediction of SNVs at each position averaged over strains was 99.58%. The run used was the one with $G = 5$ and lowest posterior mean deviance. The haplotype predictions were taken as the posterior mean of the $\tau_{v,g,a}$ discretised by setting $\tau_{v,g,m} = 1$ and $\tau_{v,g,a \neq m} = 0$ where $m = \arg\max_a \tau_{v,g,a}$.

Table S5: Comparison of inferred strains to true reference genomes in the 'strain' mock using the `Lineage` algorithm [4] for 1,000 variant positions.

	Strain genomes				
Predicted	EC_O127	EC_O104	EC_O157	EC_K12	EC_UT189
H_0	147	741	740	736	138
H_1	600	243	333	0	631
H_2	600	243	333	0	631
H_3	705	405	162	177	726
H_4	601	7	354	236	632

This table compares the predicted strains for one run of the `Lineage` algorithm with the five known *E. coli* genomes at each of 1,000 variant positions chosen at random from the 6,044 variant positions. The integer frequencies give the number of positions where each strain (row) differs from a genome (column). The overall accuracy rate for mapping of strains onto genomes was 76.32%.

Table S6: Comparison of inferred strains to true reference genomes in the 'strain' mock using the `DESMAN` algorithm for 1,000 variant positions.

	Strain genomes				
Predicted	EC_O127	EC_O104	EC_O157	EC_K12	EC_UT189
H_0	600	243	334	0	634
H_1	629	359	9	331	643
H_2	277	635	646	631	5
H_3	2	604	628	598	282
H_4	602	4	355	239	636

This table compares the predicted strains for one run of the `DESMAN` algorithm with the five known *E. coli* genomes at each of 1,000 variant positions chosen at random from the 6,044 variant positions. The integer frequencies give the number of positions where each strain (row) differs from a genome (column). The overall SNV accuracy rate for mapping of strains onto genomes was 99.6%. The run used was the one with $G = 5$ and lowest posterior mean deviance. The haplotype predictions were taken as the discretised posterior mean of the $\tau_{v,g,a}$.

Table S7: Closest matching reference genomes for the eight inferred STEC strains from *E. coli* O104:H4 outbreak.

Strain	Closest ref.	d	1.0 - d
H_0	E. coli 536	0.00758	0.99242
H_1	E. coli O127 H6 E2348	0.02117	0.97883
H_2	E. coli APEC O78	0.00305	0.99695
H_3	E. coli UMN026	0.04244	0.95756
H_4	E. coli APEC O78	0.02039	0.97961
H_5	E. coli ABU 83972	0.00195	0.99805
H_6	E. coli APEC O78	0.01411	0.98589
H_7	E. coli O104 H4 2011C	0.00165	0.99835

Inferred strain sequences were aligned with references and a phylogenetic tree constructed for the eight inferred strains found for the *E. coli* O104:H4 outbreak. The run used was the one lowest posterior mean deviance. The haplotype predictions were taken as the discretised posterior mean of the $\tau_{v,g,a}$. The SCSGs for the strains and reference genomes were aligned separately using `mafft`[5], trimmed and then concatenated together. The tree was constructed using `FastTree`[6]. Here we give for each strain the closest reference in terms of phylogenetic distance, d, or total average nucleotide substitutions.

Table S8: Comparison of true strains to `DESMAN` and `Lineage` inferred haplotypes for 25 clusters in the 'complex strain' mock.

$Cluster$	$TaxaID$	Cov	V	H	G	G'	Err	Err'
Cluster3	75985	220	54	4	4	3	0.016	0.0142
Cluster5	2209	87	185	5	2	2	0.328	0.358
Cluster9	777	270	83	3	3	3	0	0
Cluster22	34085	310	210	4	3	3	0.0111	0.00952
Cluster24	774	1400	151	4	4	5	0.00662	0.2
Cluster31	948	530	10	4	4	3	0	0
Cluster32	285	240	244	2	2	2	0.0372	0.0331
Cluster37	1833	300	99	3	3	3	0	0.0034
Cluster38	714	290	189	4	3	2	0.03	0.0556
Cluster39	1509	150	234	2	2	2	0.00214	0.00641
Cluster44	480	630	54	3	3	3	0	0
Cluster56	28173	190	206	2	2	2	0.00245	0
Cluster58	96345	100	57	3	2	2	0.00877	0.00877
Cluster59	587753	310	303	4	4	4	0.0347	0.0382
Cluster64	2162	710	70	2	2	2	0	0
Cluster67	28108	130	147	5	3	3	0.0208	0.0185
Cluster72	382	720	42	2	2	2	0	0
Cluster76	29447	500	170	5	4	3	0.0368	0.049
Cluster78	1402	3400	95	2	2	2	0	0
Cluster83	715	260	147	3	3	3	0.0403	0.0349
Cluster86	1502	150	86	2	2	2	0	0
Cluster90	85698	90	133	2	2	2	0	0
Cluster101	1744	140	68	2	2	2	0	0
Cluster104	1681	190	196	5	2	3	0.0204	0.0663
Cluster132	2095	420	18	2	2	2	0	0

For the 25 75% complete `CONCOCT` clusters that possessed at least five variants and mapped onto species with strain variation, we give the NCBI Taxa ID that the cluster mapped to $TaxaID$, the total coverage summed across all 96 samples Cov, the number of variant positions predicted on the SCGs V, the true number of strains H, the inferred number of strains G by `DESMAN`, the number G' inferred by the `Lineage` algorithm, the mean error rate in SCG SNV predictions averaged over strains (Err) from `DESMAN` and mean error rate for `Lineage` (Err').

Table S9: Summary of the Tara regional co-assemblies.

Name	Acronym	No. of metagenomes	Reads(billion)	Contigs >2.5Kbp	Contig length >2.5Kbp (Mbp)
Atlantic Ocean (North East)	ANE	7	2.47	250,172	1,261
Atlantic Ocean (North West)	ANW	6	1.92	223,940	1,144
Atlantic Ocean (South East)	ASE	6	1.23	107,759	493
Atlantic Ocean (South West)	ASW	6	2.17	158,640	781
Indian Ocean (North)	ION	11	3.90	272,745	1,322
Indian Ocean (South)	IOS	10	3.41	229,550	1,072
Mediterranean Sea	MED	7	2.37	243,494	1,357
Pacific Ocean (North)	PON	8	2.87	279,231	1,427
Pacific Ocean (South East)	PSE	16	5.33	449,817	2,260
Pacific Ocean (South West)	PSW	7	2.16	203,431	1,142
Red Sea	RED	6	1.93	205,415	1,144
Southern Ocean	SOC	3	1.14	19,160 *	185

The MAG name is the same as used in the original publication by Delmont *et al.* [1].

Table S10: Summary of results from applying the DESMAN pipeline to the 32 Tara MAGs with coverage > 100.

Name	Phylum	Length	NM	S	SCG	$FSCG$	C	SNV	F	tf	vf	G	Err
TARA_ANE_MAG_00002	Proteobacteria	3133556	47	18	36	36	17453	91	0.52	0.42	0.56	1	0.42
TARA_ANE_MAG_00018	Proteobacteria	1079517	19	19	33	27	9636	145	1.5	0.64	0.56	2	0.64
TARA_ANE_MAG_00026	Bacteroidetes	1357143	21	27	35	25	19461	962	4.94	0.69	0.59	2	0.69
TARA_ANE_MAG_00032	Proteobacteria	1808585	38	14	28	18	6238	104	1.67	0.43	0.38	2	0.43
TARA_ANE_MAG_00050	Proteobacteria	958701	21	16	28	24	13985	918	6.56	0.61	0.59	3	0.61
TARA_ANW_MAG_00006	Proteobacteria	5485499	70	25	36	27	22761	84	0.37	0.46	0.63	2	0.46
TARA_ANW_MAG_00043	Euryarchaeota	1179335	9	14	28	21	13034	408	3.13	0.52	0.5	3	0.52
TARA_ION_MAG_00039	Bacteroidetes	1530244	23	19	28	22	11341	332	2.93	0.68	0.57	2	0.68
TARA_ION_MAG_00055	Actinobacteria	1230383	13	19	30	21	6127	97	1.58	0.46	0.47	2	0.46
TARA_IOS_MAG_00011	Actinobacteria	1769542	33	11	34	19	673	1	0.15	0.59	0	1	0.59
TARA_IOS_MAG_00024	Proteobacteria	895557	30	21	34	23	13063	364	2.79	0.71	0.6	2	0.71
TARA_IOS_MAG_00045	Euryarchaeota	1184019	13	13	31	25	14426	354	2.45	0.5	0.46	3	0.5
TARA_IOS_MAG_00046	Actinobacteria	1601565	24	20	31	23	7116	92	1.29	0.47	0.57	2	0.47
TARA_MED_MAG_00026	Proteobacteria	1378810	33	27	34	21	8130	97	1.19	0.64	0.6	2	0.64
TARA_MED_MAG_00040	C. Marinimicrobia	1716686	13	47	34	31	21131	2602	12.31	0.65	0.6	3	0.65
TARA_MED_MAG_00051	Proteobacteria	967888	37	19	34	26	11276	247	2.19	0.68	0.5	2	0.68
TARA_MED_MAG_00062	Bacteroidetes	1283464	26	19	32	24	16260	1852	11.39	0.62	0.56	5	0.62
TARA_MED_MAG_00070	Proteobacteria	886127	24	23	33	21	12489	148	1.19	0.68	0.55	3	0.68
TARA_MED_MAG_00092	Proteobacteria	1407426	21	20	28	19	10431	520	4.99	0.64	0.54	2	0.64
TARA_MED_MAG_00093	Bacteroidetes	1206176	26	12	31	28	21970	1763	8.02	0.68	0.6	3	0.68
TARA_MED_MAG_00101	Bacteroidetes	1246628	17	12	29	22	12167	417	3.43	0.65	0.58	3	0.65
TARA_MED_MAG_00110	Proteobacteria	890789	10	54	28	20	15456	1943	12.57	0.64	0.59	3	0.64
TARA_PON_MAG_00026	Proteobacteria	2180211	23	25	34	33	25359	1437	5.67	0.52	0.5	2	0.52
TARA_PSE_MAG_00001	Bacteroidetes	3950680	49	22	36	31	25481	18	0.07	0.57	0.67	1	0.57
TARA_PSW_MAG_00048	Proteobacteria	1681719	44	25	33	26	19549	1073	5.49	0.68	0.63	2	0.68
TARA_PSW_MAG_00066	Actinobacteria	2033721	27	18	33	19	11523	118	1.02	0.47	0.32	2	0.47
TARA_PSW_MAG_00074	Proteobacteria	1189144	31	41	28	21	14704	563	3.83	0.67	0.57	3	0.67
TARA_PSW_MAG_00078	Proteobacteria	670241	13	14	30	22	13582	636	4.68	0.65	0.6	2	0.65
TARA_PSW_MAG_00081	Chloroflexi	934890	20	29	30	26	16979	584	3.44	0.67	0.56	4	0.67
TARA_RED_MAG_00001	Proteobacteria	2849757	48	16	36	31	12671	135	1.07	0.35	0.36	2	0.35
TARA_RED_MAG_00009	Proteobacteria	930732	28	25	36	26	12293	388	3.16	0.69	0.6	3	0.69
TARA_RED_MAG_00062	Euryarchaeota	1266249	13	21	28	19	7914	115	1.45	0.61	0.57	2	0.61

The MAG name is the same as used in the original publication by Delmont *et al.* [1]. Phylum is the taxonomic assignment from that paper. Length is the total MAG sequence length in bp. NM gives the number of KEGG Pathway modules assigned to the MAG. S the number of metagenome samples for which the coverage was > 1.0. SCG the number of single copy core genes (SCGs) in the MAG. $FSCG$ the number after filtering based on outlying sample coverages. C the bp of genes used for variant detection. SNV the number of variants detected. F the fraction of variable bases. tf the fraction of AT bases in the non-variable positions, vf the fraction of AT bases in the variable positions. G the number of inferred haplotypes and Err the estimated percentage uncertainty in those inferred haplotypes.

References

[1] Delmont, T.O., Quince, C., Shaiber, A., Esen, O.C., Lee, S.T.M., Lucker, S., Eren, A.M.: Nitrogen-fixing populations of Planctomycetes and Proteobacteria are abundant in the surface ocean. http://biorxiv.org/content/early/2017/04/23/12979

[2] Boisvert, S., Raymond, F., Godzaridis, E., Laviolette, F., Corbeil, J.: Ray Meta: scalable de novo metagenome assembly and profiling. Genome Biol. **13:R122** (2012)

[3] Dinghua, L., Chi-Man, L., Luo, R., Sadakane, K., Lam, T.-W.: Megahit: an ultra-fast single-node solution for large and complex metagenomics assembly via succinct de bruijn graph. Bioinformatics **31: 1674-1676** (2015)

[4] O'Brien, J.D., Didelot, X., Iqbal, Z., Amenga-Etego, L., Ahiska, B., Falush, D.: A bayesian approach to inferring the phylogenetic structure of communities from metagenomic data. Genetics **3:925-37** (2014)

[5] Katoh, M., Kuma, M.: Mafft: a novel method for rapid multiple sequence alignment based on fast fourier transform. Nucleic Acids Res. **30**, 3059–3066 (2002)

[6] Price, M.N., Dehal, P.S., Arkin, A.P.: Fasttree 2 approximately maximum-likelihood trees for large alignments. PLoS ONE **5**, 9490 (2010)

[7] Alneberg, J., Bjarnason, B., de Bruijn, I., Schirmer, M., Quick, J., Ijaz, U., Lahti, L., Loman, N.J., Anderson, A.F., Quince, C.: Binning metagenomic contigs by coverage and composition. Nat. Methods **11**, 1144–1146 (2014)

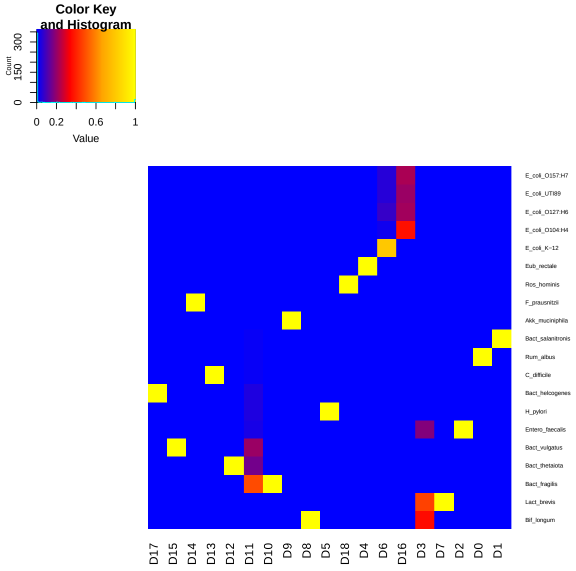

Figure S1: Confusion matrix for the synthetic 'strain' mock. Heat-map where intensity indicates proportion of contigs in each CONCOCT cluster that derive from each strain. There were 19 clusters and 20 strains. The recall was 98.1% and precision 96.1% comparing the genome labels and clusters [7] with an overall accuracy of 0.971 as given by the adjusted Rand index.

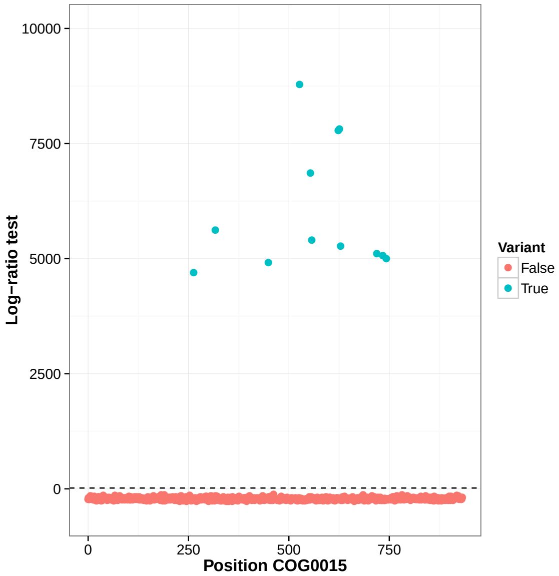

Figure S2: Log-ratio test statistic for variants on COG0015 of the synthetic 'strain' mock. The likelihood ratio test statistic (Equation 2) for positions along COG0015 - Adenylosuccinate lyase. Positions that are true variants are coloured blue, positions with no variation, red. The dashed line corresponds to a FDR or q-value of 1.0e-3. Positions above this line are classified as variants under the test. Note negative log-ratios occur because the minimum variant frequency p_L is set at 1%.

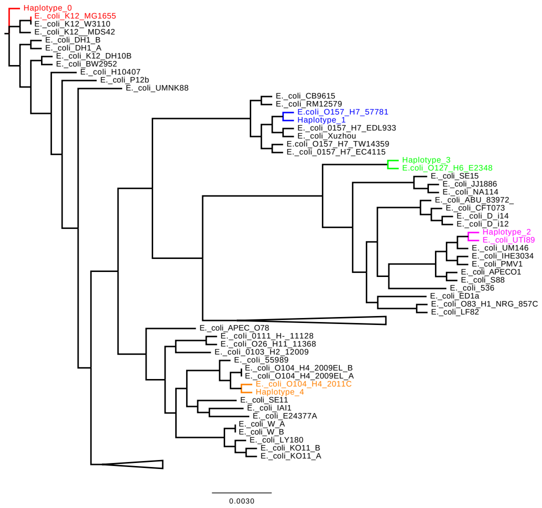

Figure S3: Phylogenetic tree constructed for the five inferred strains found in the 'synthetic strain' mock and 62 *E. coli* reference sequences. The 372 SCSGs for the strains and reference genomes were aligned separately using `mafft`[5], trimmed and then concatenated together. The tree was constructed using `FastTree`[6]. The known reference genome each strain mapped onto from Supplementary Table S4 is shown in the same colour as the corresponding strain. These results were for the run with $G = 5$ that had the lowest posterior mean deviance.

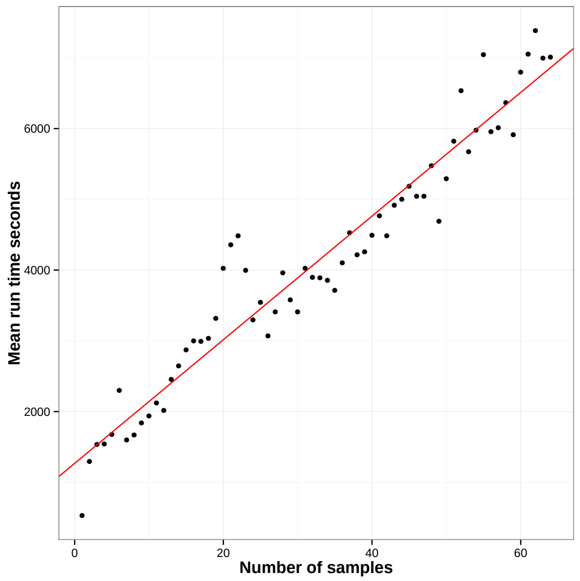

Figure S4: Run times for `DESMAN` in seconds for increasing sample number. The total run time for `DESMAN` on the synthetic 'strain' mock is shown as a function of number of samples. Results are the mean time averaged over twenty replicates comprising random subsamples of the original 64 samples. Each run was for $G = 5$ and run on a Intel(R) Xeon(R) CPU E7-8850 v2 @ 2.30GHz.

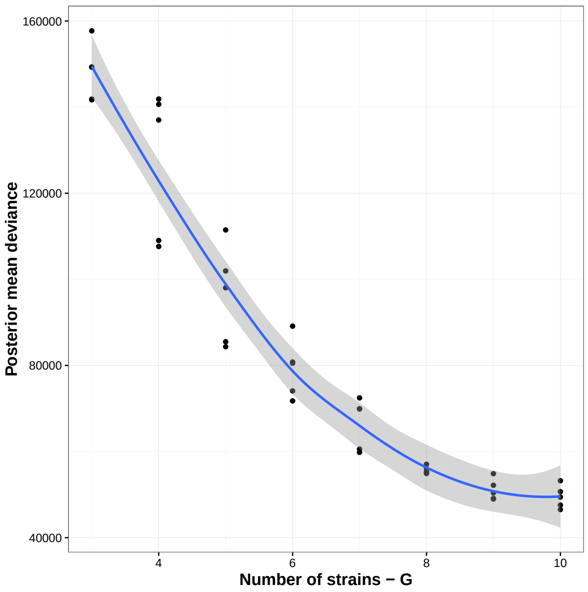

Figure S5: Posterior mean deviance as a function of *G* for the *E. coli* O104:H4 outbreak SCSG positions. The posterior mean deviance as a function of G for five replicates running the strain resolution Gibbs sampler for 1,000 random positions from the 28,435 potential variants identified on the 440 SCSGs for the *E. coli* clusters in the *E. coli* O104:H4 outbreak.

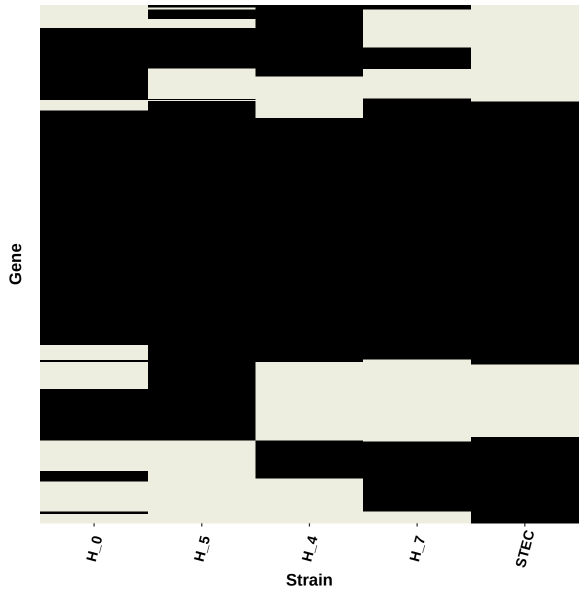

Figure S6: Comparison of gene presence/absence inferred for the four strains we are confident in from the STEC *E. coli* O104:H4 outbreak together with results for the known outbreak genome. Gene presence/absences (black/ivory) were inferred for the strains. They were calculated for the STEC genome (*Escherichia coli* O104:H4 str. 2011C) by mapping using `MUMmer`. Comparing H_7 and the STEC genome, 91.8% of counts matched. These results were for the run with $G = 8$ that had the lowest posterior mean deviance.

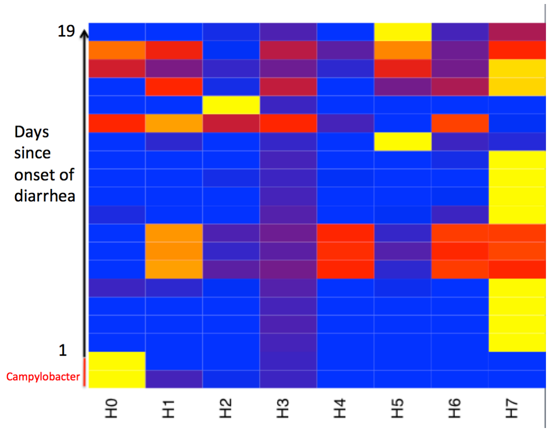

Figure S7: strain relative abundances across samples for the STEC *E. coli* O104:H4 outbreak. The relative frequencies of the eight predicted *E. coli* strains in the twenty samples from the STEC *E. coli* O104:H4 outbreak that had a mean coverage across *E. coli* SCSGs greater than five. The two samples at the bottom of the heat map derived from a *Campylobacter jejuni* infected individual, the other 18 samples were all determined to be infected with STEC. We have ordered these by 'Days since onset of diarrhea (ddays)', the number of days ago that the individual first experienced diarrhea symptoms. The relative abundance of strain H7 negatively correlates with *ddays* ($\tau = -0.366$, $p = 0.0414$, Kendall's tau coefficient).

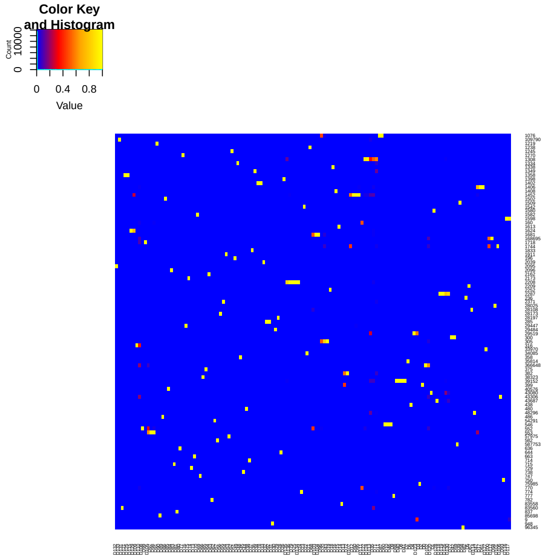

Figure S8: Confusion matrix for the synthetic 'complex strain' mock. Heatmap where intensity indicates proportion of contigs in each CONCOCT cluster that derive from each species. There were 137 clusters and 100 species. The recall was 86.1% and precision 98.2% comparing the species labels and clusters [7] with an overall accuracy of 0.830 as given by the adjusted Rand index.

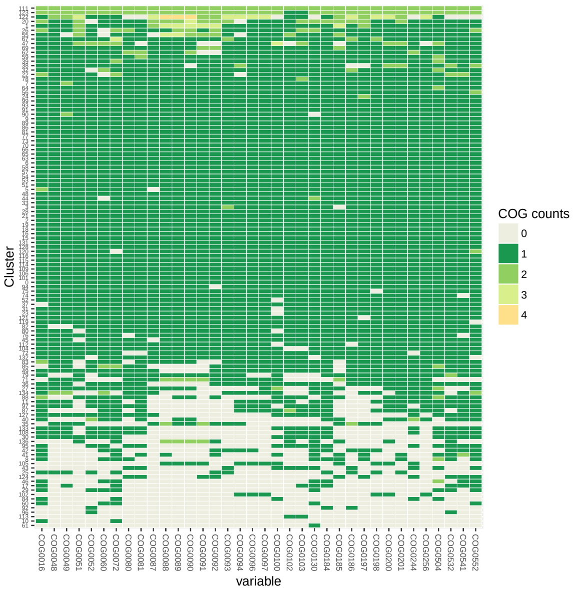

Figure S9: Single core gene frequencies in the 137 `CONCOCT` clusters generated by binning all contigs $> 1kbp$ in length from the 96 'complex strain' mock samples. 75 of these clusters were at least 75% pure and complete. Each row corresponds to a cluster and columns are SCGs.

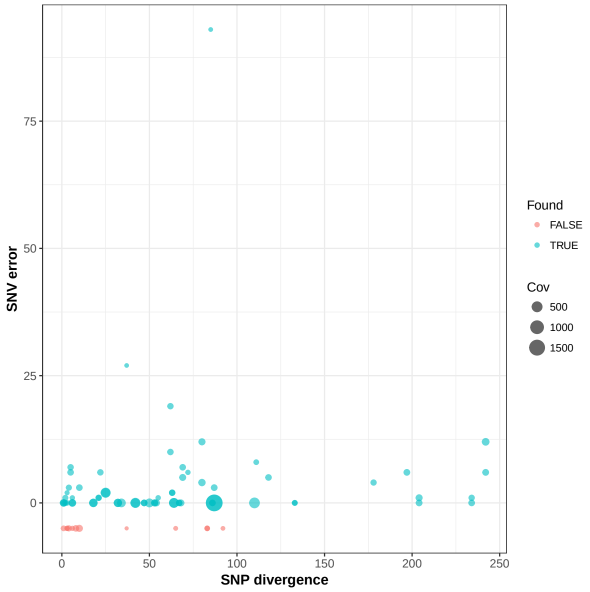

Figure S10: Number of SNV errors in inferred haplotypes vs. the number of SNP differences to closest matching reference. For every reference strain in the 25 species that mapped onto the 25 clusters for which multiple strains were present and at least five variants were observed (79 in total) we calculated the SNP divergence to the most similar strain in that species (x-axis, S). Then for the 67 strains (84.8%) that were detected as haplotypes by `DESMAN` we calculated their error rate as SNVs different to the true reference strain (y-axis, E). Those strains not found are placed on the negative y-axis. There was a negative correlation between fractional error rate ($f = E/S$) and SNP divergence (S) (p-value=0.036) for those strains that were detected. There was a positive correlation between individual strain coverage and the probability that a strain is actually detected (logistic regression - estimate 0.043 ± 0.015, p-value = 0.00352).

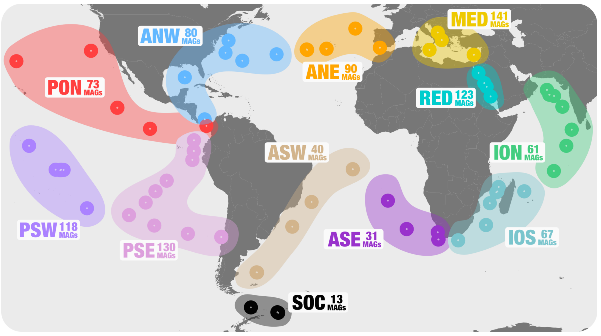

Figure S11: Tara MAG summary. Summary showing the 12 geographic regions corresponding to the co-assemblies together with the number of MAGs obtained from each. Reproduced from Delmont *et al.* [1].

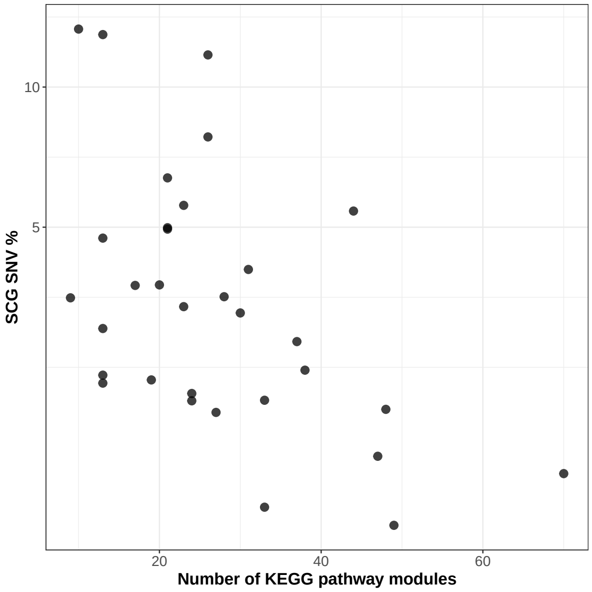

Figure S12: Top panel) SCG SNV frequency as a percentage against number of KEGG Pathway modules for the 32 Tara MAGs with total coverage > 100. A significant negative correlation was observed (Spearman's test, $\rho = -0.485$, p-value $= 0.0049$).

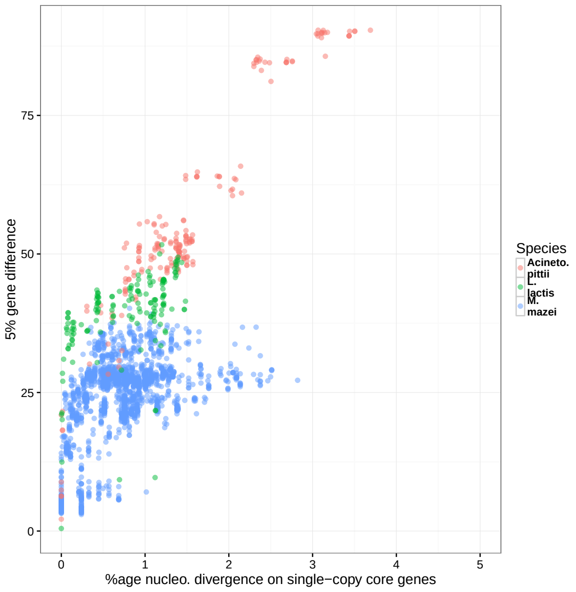

Figure S13: Percentage SCG nucleotide divergence against percentage genome divergence for strains deriving from three environmental species. For each species (*Methanosarcina mazei*, *Lactococcus lactis* and *Acinetobacter pittii*), strains were downloaded from the NCBI list of bacterial and archaeal genomes. We then compared percentage nucleotide divergence on the single-copy core genes with genome divergence as a percentage of 5% gene clusters not shared between each pair of strains with nucleotide divergence $< 5\%$ for each species. In all three cases the relationship was highly significant but with different regression coefficients (*Methanosarcina mazei* — coeff. 7.7, Adjusted R-squared: 0.2246, p-value $<$ 2.2e-16, *Lactococcus lactis* — coeff. 8.2, Adjusted R-squared: 0.2401, p-value = 3.829e-11 and *Acinetobacter pittii* — coeff. 21.4, Adjusted R-squared: 0.8892, p-value $<$ 2.2e-16).
